# Hemi-methylated DNA opens a closed conformation of UHRF1 to facilitate its histone recognition

**DOI:** 10.1038/ncomms11197

**Published:** 2016-04-05

**Authors:** Jian Fang, Jingdong Cheng, Jiaolong Wang, Qiao Zhang, Mengjie Liu, Rui Gong, Ping Wang, Xiaodan Zhang, Yangyang Feng, Wenxian Lan, Zhou Gong, Chun Tang, Jiemin Wong, Huirong Yang, Chunyang Cao, Yanhui Xu

**Affiliations:** 1 Key Laboratory of Molecular Medicine, Ministry of Education, Department of Systems Biology for Medicine, School of Basic Medical Sciences, Shanghai Medical College of Fudan University, Shanghai 200032, China; 2 State Key Laboratory of Genetic Engineering, Collaborative Innovation Center of Genetics and Development, School of Life Sciences, Fudan University, Shanghai 200433, China; 3 Fudan University Shanghai Cancer Center, Institute of Biomedical Sciences, Shanghai Medical College of Fudan University, Shanghai 200032, China; 4 State Key Laboratory of Bio-organic and Natural Product Chemistry, Shanghai Institute of Organic Chemistry, Chinese Academy of Sciences, 345 LingLing Road, Shanghai 200032, China; 5 Shanghai Key Laboratory of Regulatory Biology, Institute of Biomedical Sciences and School of Life Sciences, East China Normal University, Shanghai 200241, China; 6 State Key Laboratory of Magnetic Resonance and Atomic and Molecular Physics, Wuhan Institute of Physics and Mathematics, Chinese Academy of Sciences, Wuhan 430071, China

## Abstract

UHRF1 is an important epigenetic regulator for maintenance DNA methylation. UHRF1 recognizes hemi-methylated DNA (hm-DNA) and trimethylation of histone H3K9 (H3K9me3), but the regulatory mechanism remains unknown. Here we show that UHRF1 adopts a closed conformation, in which a C-terminal region (Spacer) binds to the tandem Tudor domain (TTD) and inhibits H3K9me3 recognition, whereas the SET-and-RING-associated (SRA) domain binds to the plant homeodomain (PHD) and inhibits H3R2 recognition. Hm-DNA impairs the intramolecular interactions and promotes H3K9me3 recognition by TTD–PHD. The Spacer also facilitates UHRF1–DNMT1 interaction and enhances hm-DNA-binding affinity of the SRA. When TTD–PHD binds to H3K9me3, SRA-Spacer may exist in a dynamic equilibrium: either recognizes hm-DNA or recruits DNMT1 to chromatin. Our study reveals the mechanism for regulation of H3K9me3 and hm-DNA recognition by URHF1.

## Abstract (web summary)

UHRF1 is involved in the maintenance of DNA methylation, but the regulatory mechanism of this epigenetic regulator is unclear. Here, the authors show that it has a closed conformation and are able to make conclusions about the mechanism of recognition of epigenetic marks.

## Graphical abstract

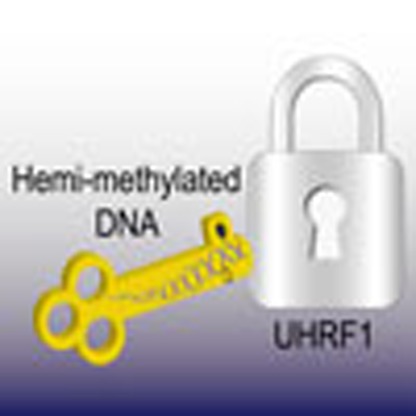

DNA methylation is an important epigenetic modification for gene repression, X-chromosome inactivation, genome imprinting and maintenance of genome stability^1^,^2^,^3^,^4^,^5^. Mammalian DNA methylation is established by *de novo* DNA methyltransferases DNMT3A/3B, and DNA methylation patterns are maintained by maintenance DNA methyltransferase 1 (DNMT1) during DNA replication^6^,^7^,^8^. Ubiquitin-like, containing PHD and RING fingers domains, 1 (UHRF1, also known as ICBP90 and NP95 in mouse) was shown to be essential for maintenance DNA methylation through recruiting DNMT1 to replication forks in S phase of the cell cycle^9^,^10^. UHRF1 is essential for S phase entry^11^,^12^ and is involved in heterochromatin formation^13^. UHRF1 also plays an important role in promoting proliferation and is shown to be upregulated in a number of cancers, suggesting that UHRF1 may serve as a potential drug target for therapeutic applications^14^,^15^,^16^,^17^.

UHRF1 is a multi-domain containing protein connecting histone modification and DNA methylation. As shown in Fig. 1a, UHRF1 is comprised of an N-terminal ubiquitin-like domain, followed by a tandem Tudor domain (TTD containing TTD^N^ and TTD^C^ sub-domains), a plant homeodomain (PHD), a SET-and-RING-associated (SRA) domain, and a C-terminal really interesting new gene (RING) domain. We and other groups demonstrated that the TTD and the PHD coordinately recognize histone H3K9me3, in which residue R2 is recognized by the PHD and tri-methylation of residue K9 (K9me3) is recognized by the TTD^18^,^19^,^20^,^21^,^22^,^23^,^24^. The SRA preferentially binds to hemi-methylated DNA (hm-DNA)^25^,^26^,^27^. Recent studies show that the SRA directly binds to replication focus targeting sequence (RFTS) of DNMT1 (RFTS^DNMT1^)^28^,^29^,^30^. A spacer region (Fig. 1a, designated Spacer hereafter) connecting the SRA and the RING is rich in basic residues and predicted to be unstructured for unknown function. Recent study shows that phosphatidylinostiol phosphate PI5P binds to the Spacer and induces a conformational change of UHRF1 to allow the TTD to recognize H3K9me3 (ref. 31). These studies indicate that UHRF1 connects dynamic regulation of DNA methylation and H3K9me3, which are positively correlated in human genome. However, how UHRF1 regulates the recognition of these two repressive epigenetic marks and recruits DNMT1 for chromatin localization remain largely unknown.

Here we report that UHRF1 adopts a closed conformation, in which the C-terminal Spacer binds to the TTD and inhibits its recognition of H3K9me3, whereas the SRA binds to the PHD and inhibits its recognition of H3R2 (unmethylated histone H3 at residue R2). Upon binding to hm-DNA, UHRF1 impairs the intramolecular interactions and promotes the H3K9me3 recognition by TTD–PHD, which may further enhance its genomic localization. As a result, UHRF1 is locked in the open conformation by the association of H3K9me3 by TTD–PHD, and thus SRA-Spacer either recognizes hm-DNA or recruits DNMT1 for DNA methylation. Therefore, UHRF1 may engage in a sophisticated regulation for its chromatin localization and recruitment of DNMT1 through a mechanism yet to be fully elucidated. Our study reveals the mechanism for regulation of H3K9me3 and hm-DNA recognition by UHRF1.

## Results

### Hm-DNA facilitates histone H3K9me3 recognition by UHRF1

**Figure 1:**
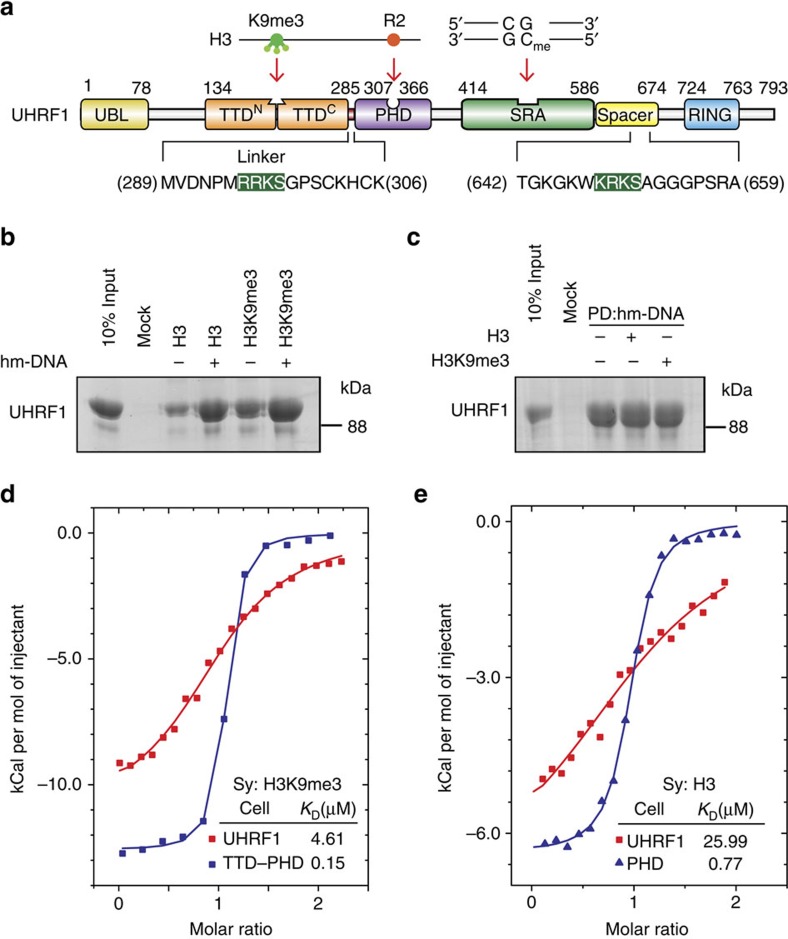
Hm-DNA facilities histione tails recognition by full-length UHRF1. (**a**) Colour-coded domain structure of human UHRF1. The boundaries of the domains are indicated with the numbers representing the amino-acid positions. Note that the conserved motif (green background) of the Linker (residues 286–306) and the Spacer (residues 587–674) bind to the TTD in a similar manner (Fig. 3b). (**b**) Hm-DNA facilities histone H3 and H3K9me3 recognition by UHRF1. Purified full-length UHRF1 was incubated with biotinylated H3 (1–21) or H3K9me3 (1–21) peptides in the presence or absence of hm-DNA (molar ratio UHRF1/hm-DNA=1:2). The mixture was immobilized onto streptavidin Sepharose beads. The bound proteins were analysed in SDS–PAGE followed by Coomassie blue staining. Sequences of the peptides are indicated in Supplementary Table 1. (**c**) Histone peptides do not affect hm-DNA-binding affinity of UHRF1. Full-length UHRF1 was incubated with biotinylated hm-DNA in the presence or absence of H3 (1–17) or H3K9me3 (1–17) peptides and analysed as in **b**. (**d**,**e**) Superimposed ITC enthalpy plots for binding of H3K9me3 peptide (1–17) to TTD–PHD and full-length UHRF1 (**d**), and H3 peptide (1–17) to the PHD and full-length UHRF1 (**e**). The estimated binding affinities (*K*_D_) are listed. The samples in the syringe (designated Sy hereafter) and cell are indicated.

To investigate how UHRF1 coordinates the recognition of H3K9me3 and hm-DNA, we purified recombinant UHRF1 (truncations and mutations) proteins from bacteria. We first performed an *in vitro* pull-down assay using biotinylated histone H3 peptides and hm-DNA (Supplementary Table 1). As shown in Fig. 1b, hm-DNA largely enhanced the interaction between full-length UHRF1 and unmethylated histone H3 (H3K9me0) or H3K9me3 peptide. Compared with hm-DNA, um-DNA (unmethylated DNA) or fm-DNA (fully methylated DNA) showed marginal effect on facilitating the interaction between UHRF1 and histone peptides, which is consistent with previous studies that UHRF1 prefers hm-DNA for chromatin association (Supplementary Fig. 1a)^25^,^26^,^27^. In contrast, histone peptides showed no enhancement on the interaction between hm-DNA and UHRF1 (Fig. 1c). These results suggest that hm-DNA facilitates histone recognition by UHRF1.

Our previous studies show that the PHD recognizes H3K9me0 and the TTD and the PHD together (TTD–PHD) coordinately recognize H3K9me3 (refs. 19, 20). We noticed that the isolated TTD–PHD showed much higher (∼31-fold) binding affinity to H3K9me3 peptide than full-length UHRF1 (Fig. 1d and Supplementary Table 2), and the isolated PHD showed much higher (∼34-fold) binding affinity to H3K9me0 peptide than full-length UHRF1 (Fig. 1e). The gel filtration analysis showed that UHRF1 is a monomer in solution (Supplementary Fig. 1b), indicating that the intramolecular (not intermolecular) interaction of UHRF1 regulates histone recognition. These results suggest that UHRF1 adopts an unfavourable conformation for histone H3 tails recognition, in which TTD–PHD might be blocked by other regions of UHRF1, and hm-DNA impairs this intramolecular interaction to facilitate its recognition of histone H3 tails.

### Intramolecular interaction within UHRF1

**Figure 2:**
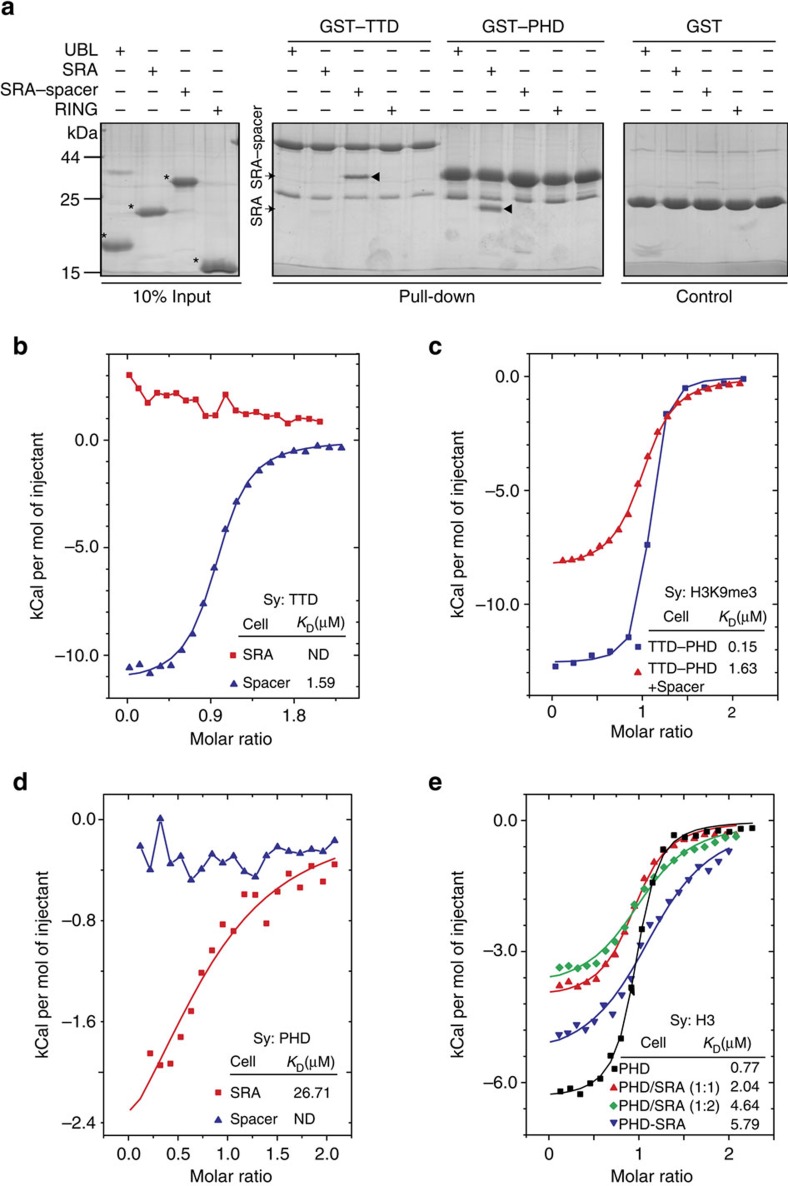
Intramolecular interactions inhibit histone recognition by UHRF1. (**a**) GST pull-down assays for the intramolecular interactions. The isolated domains of UHRF1 were incubated with GST-tagged TTD or PHD immobilized on glutathione resin. The bound proteins were analysed by SDS–PAGE and Coomassie blue staining. (**b**,**d**) Superimposed ITC enthalpy plots for the intramolecular interactions of isolated UHRF1 domains. The estimated binding affinities (*K*_D_) were listed. ND, not detectable. (**c**) Superimposed ITC enthalpy plots for the binding of H3K9me3 to TTD–PHD in the absence or presence of the Spacer (molar ratio TTD–PHD/Spacer=1:2). (**e**) Superimposed ITC enthalpy plots for the binding of H3 to PHD–SRA or PHD in the absence or presence of the SRA (molar ratio PHD/SRA=1:1 or 1:2). ND, not determined; Sy., syringe.

To test above hypothesis, we performed glutathione *S*-transferase (GST) pull-down assay using various truncations of UHRF1. Interestingly, the TTD directly bound to SRA-Spacer but not the SRA, suggesting that the Spacer (residues 587–674) is important for the intramolecular interaction (Fig. 2a). The isothermal titration calorimetry (ITC) measurements show that the TTD bound to the Spacer (but not the SRA) in a 1:1 stoichiometry with a binding affinity (*K*_D_) of 1.59 μM (Fig. 2b). The presence of the Spacer markedly impaired the interaction between TTD–PHD and H3K9me3 (Fig. 2c). The results indicate that the Spacer directly binds to the TTD and inhibits its interaction with H3K9me3.

The GST pull-down assay also shows that the PHD bound to the SRA, which was further confirmed by the ITC measurements (*K*_D_=26.7 μM; Fig. 2a,d). Compared with the PHD alone, PHD-SRA showed decreased binding affinity to H3K9me0 peptide by a factor of eight (Fig. 2e). Pre-incubation of the SRA also modestly impaired PHD–H3K9me0 interaction. These results indicate that the SRA directly binds to the PHD and inhibits its binding affinity to H3K9me0. Taken together, UHRF1 seems to adopt a closed form through intramolecular interactions (TTD–Spacer and PHD-SRA), which inhibit histone H3 tail recognition by UHRF1.

### Overall structure of TTD–Spacer

To investigate the intramolecular interaction within UHRF1, we first mapped the minimal regions within the Spacer for the interaction with the TTD (Supplementary Fig. 2a). Internal deletions of the Spacer, including Spacer^Δ660–664^, Spacer^Δ665–669^, Spacer^Δ670–674^ and Spacer^642–674^, bound to the TTD with comparable binding affinities to that of the Spacer, whereas Spacer^587–641^ showed no detectable interaction. Spacer^Δ642–651^, Spacer^Δ650–654^ and Spacer^Δ655–659^ also decreased binding affinities, indicating that residues 642–674 are important for TTD–Spacer interaction.

**Figure 3:**
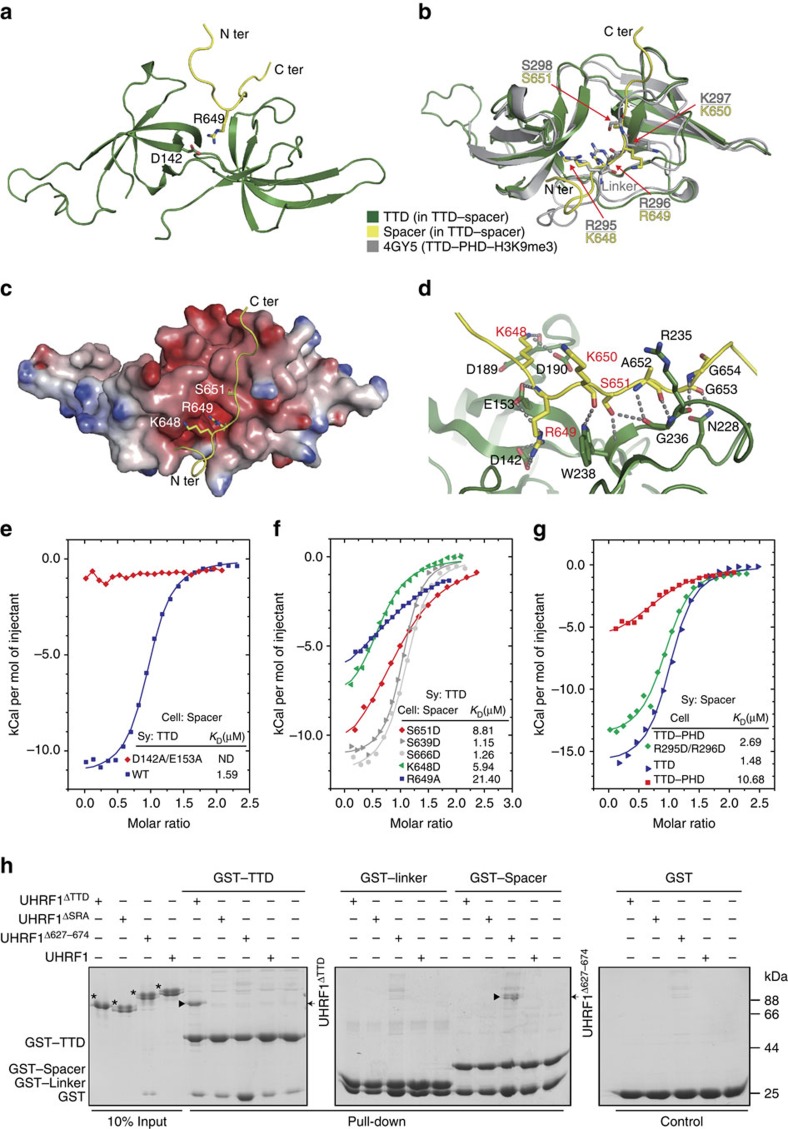
NMR structure of the TTD bound to the Spacer. (**a**) Ribbon representation of TTD–Spacer structure. N- and C-termini of the Spacer are indicated. The TTD is coloured in green, and the Spacer is coloured in yellow. The colour scheme is used the all the structure figures. (**b**) Superimposition of TTD–Spacer and TTD–PHD–H3K9me3 (4GY5.PDB) structures shown in ribbon representations. The TTD is coloured in green and the Spacer in yellow in TTD–Spacer structure. TTD–PHD–H3K9me3 complex is coloured in grey, and the PHD and H3K9me3 are omitted for simplicity. Residues for the interactions are shown in stick representation. (**c**) Electrostatic potential surface representation of the TTD with the Spacer shown in ribbon representation. The critical residues on the Spacer for the interaction are shown in stick representation. (**d**) Close-up view of TTD–Spacer interaction. Critical residues for the interaction are shown in stick representation. Hydrogen bonds are indicated as dashed lines. (**e**–**g**) Superimposed ITC enthalpy plots for the interaction between the Spacer and the TTD (or TTD–PHD) with the estimated binding affinity (*K*_D_) indicated. Wild-type and mutant proteins for the measurements are indicated. (**h**) GST pull-down assays for the intramolecular interactions. The wild-type or indicated truncations of UHRF1 were incubated with GST-tagged TTD, Linker or Spacer. The mixtures were immobilized on glutathione resin. The bound proteins were analysed by SDS–PAGE and Coomassie blue staining. ND, not determined; Sy., syringe.

We next determined the solution structure of the TTD (residues 134–285) bound to Spacer^627–674^ by conventional NMR techniques (Supplementary Table 3 and Supplementary Fig. 3a,b). In the complex structure, each Tudor domain adopts a ‘Royal' fold containing a characteristic five-stranded β-sheet and the two Tudor domains tightly pack against each other with a buried area of 573 Å^2^ (Fig. 3a). The TTD adopts similar fold to that in TTD–PHD–H3K9me3 complex structure (PDB: 4GY5)^19^ with a root-mean-square deviation of 1.09 Å for 128 Cα atoms, indicating that the Spacer does not result in obvious conformational change of the TTD (Fig. 3b). The Spacer (residues 643–655 were built in the model) adopts an extended conformation and binds to an acidic groove on the TTD (Fig. 3c).

The TTD–Spacer interaction is mediated by a number of hydrogen bonds (Fig. 3d). The side chain of residue K648 forms hydrogen bonds with the carbonyl oxygen atom of D189 and side chain of D190 of the TTD. The side chain of residue R649 packs against an acidic surface mainly formed by residues D142 and E153. Residue S651 forms hydrogen bonds with the main chain of residues G236 and W238. The interaction is further supported by hydrogen bonds formed between residues K650, A652, G653 and G654 of the Spacer and residues N228, G236 and W238 of the TTD, respectively.

In support of above structural analyses, mutation D142A/E153A of the TTD abolished its interaction with the Spacer (Fig. 3e). Mutations K648D and S651D of the Spacer decreased their binding affinities to the TTD, and mutation R649A of the Spacer showed more significant decrease (∼13-fold) in the binding affinity (Fig. 3f). As negative control, mutations S639D and S666D of the Spacer showed little effect on the interaction. Interestingly, phosphorylation at residue S651 of UHRF1 was observed in previous mass-spectrometry analyses^32^. Compared with the unmodified peptide of Spacer^642–664^, a phosphorylation at S651 markedly decreased the binding affinity to the TTD (Supplementary Fig. 2b), suggesting that the phosphorylation may regulate the intramolecular interaction within UHRF1.

### The spacer binds to the TTD by competing with the linker

Previous studies indicate that the TTD binds to a linker region connecting the TTD and PHD (residues 286–306, designated Linker, Fig. 1a), and TTD–Linker interaction is essential for H3K9me3 recognition by TTD–PHD^18^,^19^,^23^. Comparison of TTD–Spacer and TTD–PHD–H3K9me3 (PDB: 4GY5) structures indicates that the Spacer and the Linker bind to the TTD in a similar manner in the two complexes (Fig. 3b). In TTD–PHD–H3K9me3 structure, residues R295, R296 and S298 of the Linker adopt almost identical conformation to residues K648, R649 and S651 of the Spacer in TTD–Spacer structure, respectively. Similar intramolecular contacts (TTD–Linker and TTD–Spacer) were observed in the two structures (Fig. 3b,d and Supplementary Fig. 4a). Thus, the Spacer may disrupt the TTD–Linker interaction and inhibits the recognition of H3K9me3 by TTD–PHD.

To test this hypothesis, we first investigated the potential competition between the Linker and the Spacer for their interaction with the TTD. The ITC experiment shows that the Linker peptide (289–306) bound to the TTD with a binding affinity of 24.04 μM (Supplementary Fig. 4b), ∼15-fold lower than that of the Spacer peptide (*K*_D_=1.59 μM, Fig. 3e). The competitive ITC experiments show that TTD–Spacer binding affinity decreased by a factor of two in the presence of the Linker, whereas TTD–Linker interaction was abolished in the presence of the Spacer (Supplementary Fig. 4c). Compared with TTD–Spacer interaction (*K*_D_=1.48 μM), TTD–PHD decreased the binding affinity to the Spacer (*K*_D_=10.68 μM), whereas mutation R295D/R296D (within the Linker and important for TTD–Linker interaction) of TTD–PHD showed minor decrease in the binding affinity (*K*_D_=2.69 μM; Fig. 3g), indicating a competition between the Spacer and the Linker on the same binding site of the TTD. Notably, although the Linker (in the context of TTD-PHD) impairs the TTD–Spacer interaction to some extent, the isolated Spacer could still bind to TTD–PHD with moderate binding affinity (*K*_D_=10.68 μM), supporting the existence of the intramolecular interaction within UHRF1.

To test whether TTD–Spacer association exists in the context of full-length UHRF1, we used various truncations of UHRF1 in the GST pull-down assay. As indicated in Fig. 3h, full-length UHRF1 and UHRF1^ΔSRA^ showed no interaction with GST-tagged TTD, Linker or Spacer, suggesting that TTD–Spacer interaction *in-cis* within full-length UHRF1 or UHRF1^ΔSRA^ prohibits TTD–Spacer complex formation *in-trans*. In contrast, UHRF1^ΔTTD^ bound to GST-TTD, and UHRF1^Δ627–674^ bound to GST-Spacer, indicating that lack of TTD–Spacer interaction *in-cis*, TTD–Spacer complex could form *in-trans*, supporting that the TTD binds to the Spacer in the context of full-length UHRF1. Moreover, GST-Linker showed very weak if not undetectable interaction with wild-type or deletions of UHRF1, suggesting that TTD–Linker interaction is much weaker than that of TTD–Spacer. Taken together, UHRF1 adopts a closed conformation, in which the Spacer binds to the TTD through competing with the Linker, and therefore inhibits H3K9me3 recognition by UHRF1.

### The spacer inhibits H3K9me3 recognition by the isolated TTD

Our previous study indicates that H3K9me3 binds to the TTD in different manner in TTD–PHD–H3K9me3 (ref. 19) and TTD-H3K9me3 (PDB: 2L3R)^21^ structures. Because the TTD is always associated with the PHD, whether the pattern of TTD–H3K9me3 interaction exists *in vivo* remains unknown. Nevertheless, comparison of TTD–H3K9me3 and TTD–Spacer structures indicates that H3K9me3 and the Spacer overlap on the surface of the TTD (Supplementary Fig. 4d), suggesting that the Spacer might block the H3K9me3 recognition by the isolated TTD. As shown in Supplementary Fig. 4e, the Spacer inhibited TTD–H3K9me3 interaction, whereas its TTD-binding defective mutants of the Spacer or the SRA (a negative control) markedly decreased the inhibition.

We next tested whether such inhibition also occurs in the context of full-length UHRF1. Compared with full-length UHRF1, UHRF1^Δ627–674^ enhanced H3K9me3-binding affinity by a factor of four (Supplementary Fig. 4f). The restoration of H3K9me3-binding affinity is not dramatic because the PHD still binds to histone H3 in both proteins. To exclude this effect, we performed the assay using UHRF1^D334A^, which abolishes H3R2-binding affinity of the PHD^19^,^20^. UHRF1^D334A^ showed undetectable H3K9me3-binding affinity, whereas UHRF1^D334A&Δ627–674^ dramatically restored its H3K9me3-binding affinity (*K*_D_=8.69 μM; Supplementary Fig. 4f), indicating that H3K9me3 recognition by the TTD is blocked by the Spacer through competitive interaction with the TTD. Moreover, the R295D/R296D mutant of full-length UHRF1 showed decreased binding affinity to H3K9me3 (eightfold lower than wild type), suggesting that mutation of R295D/R296D favours TTD–Spacer interaction and therefore promotes UHRF1 to exhibit a more stable closed conformation (Supplementary Fig. 4g). Taken together, the Spacer binds to the TTD and inhibits H3K9me3 recognition by UHRF1 through (i) disrupting TTD–Linker interaction, which is essential for H3K9me3 recognition by TTD–PHD, (ii) prohibiting H3K9me3 binding to the isolated TTD.

### TTD–PHD–H3K9me3 complex inhibits TTD–spacer interaction

Interestingly, pre-incubation of H3K9me3 peptide completely blocked the interaction between the Spacer and the TTD alone or TTD–PHD (Supplementary Fig. 4h), whereas the presence of the Spacer partially impaired the interaction between TTD–PHD and H3K9me3 (Fig. 2c). The results are also consistent with the previous observation that the interaction between TTD–PHD and the Spacer is much weaker (*K*_D_=10.68 μM, Fig. 3g) than that between TTD–PHD and H3K9me3 (*K*_D_=0.15 μM, Fig. 1d). These results suggest that once TTD–PHD binds to H3K9me3, UHRF1 will be locked by H3K9me3 and the Spacer is unlikely to fold back for the intramolecular interaction.

### Hm-DNA disrupts intramolecular interaction within UHRF1

**Figure 4:**
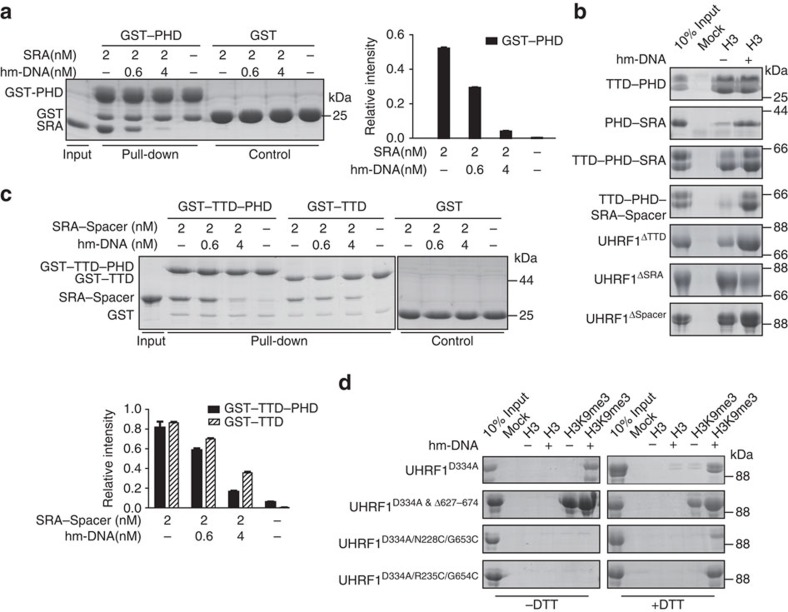
Hm-DNA impairs the intramolecular interaction of UHRF1 and facilitates its histone recognition. (**a**) Hm-DNA impairs the intramolecular interaction of PHD–SRA. The SRA was incubated with GST-tagged PHD in the presence of increasing concentrations of hm-DNA and immobilized on glutathione resin. The bound proteins were analysed in SDS–PAGE and Coomassie blue staining (left) and quantified by band densitometry (right). Error bars, s.d. for triplicate experiments. (**b**) Purified fragments of UHRF1 were analysed by histone peptide (H3K9me0) pull-down assay as described in Fig. 1b. (**c**) Hm-DNA impairs the intramolecular interaction of TTD–Spacer. SRA–Spacer was incubated with GST-tagged TTD–PHD or TTD in the presence of increasing concentrations of hm-DNA and analysed in pull-down experiment as described in **a**. The quantified band densitometries are indicated below the Coomassie blue staining. Error bars, s.d. for triplicate experiments. (**d**) Histone peptide pull-down assay using UHRF1 mutants as indicated. The assays were performed in the presence (+DTT) or absence (−DTT) of 15 mM DTT.

To investigate whether hm-DNA could open the closed conformation of UHRF1, we first measured the intramolecular interaction using UHRF1 truncations in the presence or absence of hm-DNA. The GST pull-down assays show that the PHD bound to the SRA and such interaction was impaired by the addition of hm-DNA (Fig. 4a). H3 peptide pull-down assays show that hm-DNA only enhanced the H3K9me0-binding affinities of UHRF1 truncations containing PHD-SRA, such as PHD-SRA, TTD-PHD-SRA, TTD-PHD-SRA-Spacer, UHRF1^ΔTTD^ and UHRF1^ΔSpacer^ (Fig. 4b). The result indicates that hm-DNA disrupts PHD–SRA interaction and facilitates H3K9me0-binding affinity of the PHD in a manner independent on the TTD or the Spacer.

Moreover, the TTD or TTD–PHD bound to SRA–Spacer and the interaction was impaired by the addition of hm-DNA (Fig. 4c). The ITC measurements show that the presence of hm-DNA markedly impaired the interaction between the TTD and SRA–Spacer (Supplementary Fig. 5a). However, the TTD–Spacer interaction was not affected by the presence of the hm-DNA, indicating that hm-DNA displaces the Spacer from the TTD in a SRA-dependent manner (Supplementary Fig. 5b).

To investigate whether hm-DNA disrupts TTD–Spacer interaction in the context of full-length UHRF1, we monitored the conformational changes of UHRF1 using its histone-binding affinity as read-out. UHRF1^D334A^ was used to exclude the effect of H3K9me0 recognition by the PHD. As expected, all D334A-containing mutants showed undetectable interaction with H3K9me0 (Fig. 4d). UHRF1^D334A^ bound to H3K9me3 peptide in the presence of hm-DNA, but showed no interaction in the absence of hm-DNA, which is consistent with the ITC experiments (Supplementary Fig. 4f). In contrast, UHRF1^D334A&Δ627–674^ strongly bound to H3K9me3 even in the absence of hm-DNA (Fig. 4d), indicating that the deletion of the Spacer releases otherwise blocked TTD–PHD for H3K9me3 recognition. The results further support the conclusion that the Spacer binds to the TTD in the context of full-length UHRF1 and the intramolecular interactions are disrupted by hm-DNA.

We next performed similar peptide pull-down assay using two mutants (N228C/G653C and R235C/G654C) generated on UHRF1^D334A^. Residues N228/R235 from the TTD and G653/G654 from the Spacer were chosen according to the TTD–Spacer complex structure (Supplementary Fig. 5c) so that the replaced Cysteine residues (one from the TTD and one from the Spacer) are physically close enough to each other to form a disulphide bond in the absence of reducing reagent (dithiothreitol, DTT). As shown in Fig. 4d, hm-DNA largely enhanced the H3K9me3-binding affinities of both mutants in the presence of DTT, but not in the absence of DTT, indicating that the disulphide bond formation (in the absence of DTT) disallows hm-DNA to disrupt TTD–Spacer interaction for H3K9me3 recognition. As negative controls, H3K9me3 recognition by UHRF1^D334A^ or UHRF1^D334A&Δ627–674^ is not affected by DTT.

The above results collectively demonstrate that (i) full-length UHRF1 adopts a closed form, in which the Spacer binds to the TTD and H3K9me3 recognition is inhibited; (ii) hm-DNA displaces the Spacer from the TTD in the context of full-length UHRF1 and therefore largely enhances its histone H3K9me3-binding activity in a manner independent on the PHD (SRA is required). We have previously demonstrated that hm-DNA also disrupts PHD–SRA interaction and facilitates H3K9me0-binding affinity of the PHD in a manner independent on the TTD or the Spacer. Taken together, hm-DNA disrupts the intramolecular interactions within UHRF1, and therefore facilitates the coordinate recognition of H3K9me3 by TTD–PHD.

### The spacer enhances hm-DNA-binding affinity of the SRA

**Figure 5:**
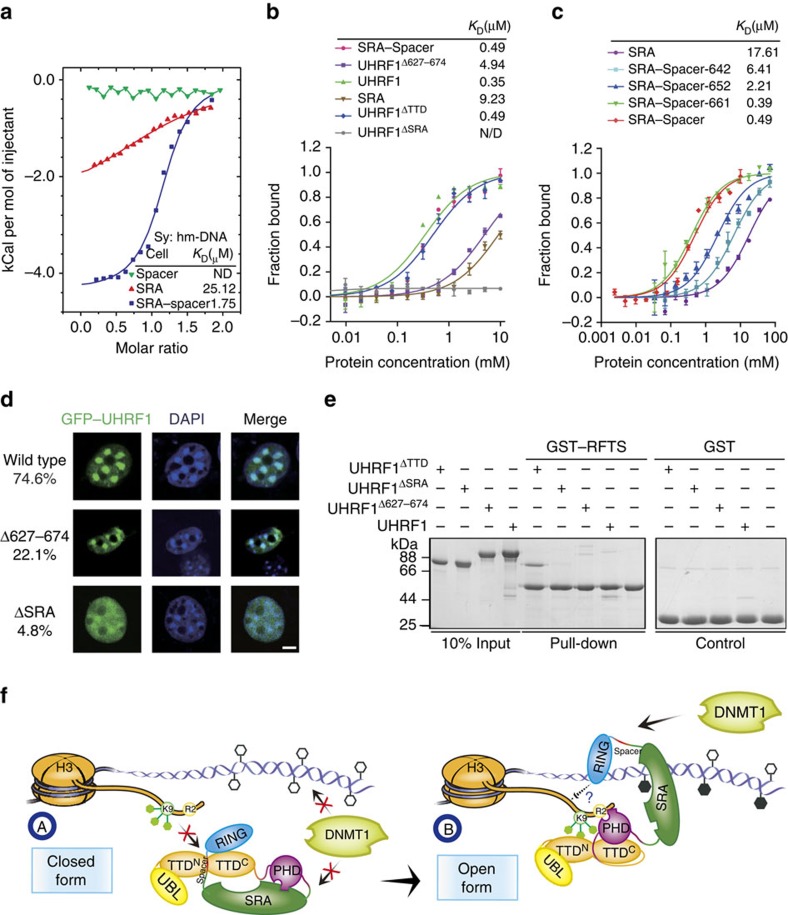
The Spacer facilitates hm-DNA–SRA interaction and DNMT1–UHRF1 interaction. (**a**) Superimposed ITC enthalpy plots for hm-DNA-binding affinities of the SRA, the Spacer and SRA–Spacer. (**b**,**c**) Superimposed fluorescence polarization (FP) plots for hm-DNA-binding affinities of truncations or full-length UHRF1. The estimated binding affinities (*K*_D_) are listed above. (**d**) Subcellular localization of GFP-tagged wild-type or indicated mutants of UHRF1 in NIH3T3 cells. The percentages of cells showing co-localization with DAPI foci were counted from at least 100 cells and shown on the left of the corresponding representative confocal microscopy. The experiment was repeated three times in the same condition. Scale bar, 5 μm. (**e**) GST pull-down experiment for the interactions between wild-type or truncations of UHRF1 and RFTS^DNMT1^ as described in Fig. 2a. (**f**) Working model for hm-DNA-mediated conformational changes of UHRF1, as described in the Discussion. ND, not determined; Sy., syringe.

To investigate how hm-DNA impairs TTD–Spacer interaction, we tested whether the Spacer is involved in hm-DNA recognition by the SRA, which is the only known domain for hm-DNA recognition within UHRF1. In the electrophoretic mobility-shift assay, SRA–Spacer showed higher hm-DNA-binding affinity than the SRA alone (Supplementary Fig. 6a). ITC measurements show that SRA–Spacer bound to hm-DNA with a much higher binding affinity (*K*_D_=1.75 μM) than the SRA (*K*_D_=25.12 μM), whereas the Spacer alone showed no interaction with hm-DNA (Fig. 5a). In the fluorescence polarization (FP) measurements, SRA–Spacer, full-length UHRF1 and UHRF1^ΔTTD^ showed comparable hm-DNA-binding affinities (Fig. 5b and Supplementary Table 4), suggesting that UHRF1 binds to hm-DNA no matter UHRF1 adopts a closed form or not. In contrast, UHRF1^ΔSRA^ abolished hm-DNA-binding affinity, indicating that the SRA is essential for hm-DNA recognition. Compared with full-length UHRF1, UHRF1^Δ627–674^ decreased the hm-DNA-binding affinity by a factor of 14 (Fig. 5b), further supporting that the Spacer plays an important role in hm-DNA recognition in the context of full-length UHRF1. In addition, hm-DNA-binding affinities of SRA or SRA–Spacer did not obviously vary upon the change of DNA lengths but did decrease with the increasing salt concentrations (Supplementary Fig. 6b,c and Supplementary Table 5). These results indicate that the Spacer not only binds to the TTD and inhibits H3K9me3 recognition when UHRF1 adopts closed conformation, but also facilitates hm-DNA recognition by the SRA when UHRF1 binds to hm-DNA.

We next mapped the minimal region of the Spacer for the enhancement of hm-DNA-binding affinity. SRA–Spacer-661 (residues 414–661) still maintained strong hm-DNA-binding affinity comparable to that of SRA–Spacer (residues 414–674), whereas SRA–Spacer-652 and SRA–Spacer-642 markedly decreased their hm-DNA-binding affinities (Fig. 5c), indicating that residues 642–661 are important for enhancing hm-DNA-binding affinity of the SRA. This minimal region largely overlaps with the Spacer region (643–655) essential for TTD interaction. We also determined the crystal structure of SRA–Spacer bound to hm-DNA at 3.15 Å resolution (Supplementary Table 6 and Supplementary Fig. 7a). The structure shows that the SRA binds to hm-DNA in a manner similar to that observed in the previously reported SRA-hm-DNA structures^18^,^26^,^27^. Intriguingly, no electron density was observed for the Spacer. A possible explanation is that the Spacer facilitates SRA–hm-DNA interaction through nonspecific salt bridge contacts because DNA is rich in acidic groups and the Spacer is rich in basic residues (Supplementary Fig. 7b). The nonspecific interaction is consistent with the previous observation that UHRF1 has no DNA sequence selectivity besides hm-CpG dinucleotide.

### The spacer is important for PCH localization of UHRF1

To investigate the role of the Spacer in the regulation of UHRF1 function, we transiently overexpressed GFP-tagged wild type or mutants of UHRF1 in NIH3T3 cells to determine their subcellular localization. For the NIH3T3 cells expressing wild-type UHRF1, most cells (∼74.6%) showed a focal pattern of protein that is co-localized with 4,6-diamidino-2-phenylindole (DAPI) foci (Fig. 5d), whereas the rest cells showed a diffuse nuclear staining pattern. The result is consistent with the previous studies that UHRF1 is mainly localized to highly methylated pericentromeric heterochromatin (PCH)^33^. In contrast, for the cells expressing UHRF1^Δ627–674^, a spacer deletion mutant with decreased hm-DNA-binding affinity (Fig. 5b), only ∼22.1% cells showed co-localization with DAPI.

Previous reports have shown that the H3K9me3 recognition of UHRF1 also plays an important role in its heterochromatin localization. For example, UHRF1 mutant (within TTD domain) lacking H3K9me3-binding affinity largely reduces its co-localization with heterochromatin^13^,^21^,^31^,^34^. Because manipulation of endogenous hm-DNA in cells is technically challenging, we used UHRF1^ΔSRA^ (lacks hm-DNA-binding affinity but maintains closed conformation, Figs 3h and 5b) to test whether closed conformation of UHRF1 exists *in vivo*. In NIH3T3 cells, UHRF1^ΔSRA^ largely decreased chromatin association (Fig. 5d). Only ∼4.8% cells expressing UHRF1^ΔSRA^ showed an intermediate enrichment, but not characteristic focal pattern, at DAPI foci, whereas the majority of the cells showed a diffuse nuclear staining pattern. The results suggest that UHRF1^ΔSRA^ adopts closed conformation so that H3K9me3 recognition by TTD–PHD is blocked by the intramolecular interaction, and support the regulatory role of the Spacer in PCH localization of UHRF1 *in vivo*.

### The spacer facilitates UHRF1–DNMT1 interaction

Previous studies show that UHRF1 recruits DNMT1 to hm-DNA for maintenance DNA methylation through the interaction between the SRA and RFTS^DNMT1^ (refs 9, 10, 28, 29, 30). We confirmed the direct interaction between RFTS^DNMT1^ and the SRA in a solution with low salt concentration (50 mM NaCl), but observed weak or undetectable interaction in a solution with higher salt concentrations (100 or 150 mM NaCl) (Supplementary Fig. 8a). Compared with the SRA, SRA–Spacer exhibited stronger interaction with RFTS^DNMT1^. In addition, RFTS^DNMT1^ bound to SRA–Spacer with a binding affinity of 7.09 μM, but showed no detectable interaction with the SRA (Supplementary Fig. 8b). Interestingly, the addition of hm-DNA abolished the interaction between RFTS^DNMT1^ and SRA–Spacer, suggesting that hm-DNA also regulates UHRF1–DNMT1 interaction (Supplementary Fig. 8c). These results indicate that the Spacer facilitates the interaction between RFTS^DNMT1^ and the SRA, and the interaction is impaired by the presence of hm-DNA.

We next tested whether the UHRF1–DNMT1 interaction is regulated by the conformational change of UHRF1. Because the addition of hm-DNA disrupts the interaction between the SRA–Spacer and RFTS^DNMT1^, we used various truncations to mimic open and closed forms of UHRF1. In the absence of hm-DNA, only UHRF1^ΔTTD^ bound to RFTS^DNMT1^, whereas full-length UHRF1, UHRF1^ΔSRA^ and UHRF1^Δ627–674^ showed undetectable interaction (Fig. 5e). As the deletion of the TTD allows UHRF1 to adopt an open conformation, the results suggest that RFTS^DNMT1^ binds to SRA–Spacer when UHRF1 adopts an open conformation in the absence of hm-DNA. In support of above observations, the addition of large amount of RFTS^DNMT1^ impaired the interaction between UHRF1 and hm-DNA (Supplementary Fig. 8d), suggesting an existence of dynamic equilibrium between UHRF1–hm-DNA and UHRF1–DNMT1 complexes.

## Discussion

According to the above results, we here proposed a working model for hm-DNA-mediated regulation of UHRF1 conformation (Fig. 5f). In the absence of hm-DNA (A), UHRF1 prefers a closed conformation, in which the Spacer binds to the TTD by competing with the Linker and the SRA binds to the PHD. As a result, the recognition of histone H3K9me3 by the TTD is blocked by the Spacer, and recognition of unmodified histone H3 (H3R2) by the PHD is inhibited by the SRA. The interaction between UHRF1 and DNMT1 is also weak because the Spacer is unable to facilitate the intermolecular interaction. In the presence of hm-DNA (B), UHRF1 prefers an open conformation, in which the SRA binds to the hm-DNA; the Spacer dissociates from the TTD and facilitates the interaction between the SRA and hm-DNA; the Linker binds to the TTD and allows TTD–PHD to recognize histone H3K9me3. When UHRF1 adopts an open conformation and has already bound to H3K9me3 (B), the interaction between H3K9me3 and TTD–PHD further prevents the Spacer from folding back to interact with the TTD, and therefore locks UHRF1 in an open conformation. The association of UHRF1 to the histone may facilitate the ubiquitination of histone tail (mediated by RING domain) for DNMT1 targeting^35^,^36^. Moreover, through a mechanism yet to be fully elucidated, DNMT1 targets hm-DNA for maintenance DNA methylation, probably through interaction with the histone ubiquitylation and/or SRA-Spacer. This cartoon summarizes our findings in this study. The *P(r)* function obtained from small-angle X-ray scattering (SAXS) measurements of TTD–PHD–SRA–Spacer–hm-DNA complex showed a broader distribution than that of the TTD–PHD–SRA–Spacer alone, supporting the proposed model that UHRF1 adopts an open conformation in the presence of hm-DNA (Supplementary Fig. 8e).

Many questions need to be further clarified. We have tried crystallizing more than three sub-constructs with and without DNA across over 1,200 crystallization conditions but failed to determine the structure of TTD–PHD–SRA–Spacer in the absence or presence of hm-DNA. Getting these structures would greatly help for understanding the hm-DNA-mediated regulation of UHRF1. In addition, this regulatory process should be further characterized using advanced techniques, such as single molecular measurement.

Our previous studies show that phosphorylation at S639 within the Spacer disrupts interaction between UHRF1 and deubiquitylase USP7 and decreases UHRF1 stability in the M phase of the cell cycle^37^. The Spacer was predicted to contain two nuclear localization signals, residues 581–600 and 648-670 (ref. 38). In this report, we found that the Spacer (i) binds to the TTD in the closed form of UHRF1 and inhibits its interaction with H3K9me3; (ii) facilitates hm-DNA recognition by the SRA and (iii) facilitates the interaction between the SRA and RFTS^DNMT1^. These findings together indicate that the Spacer plays a very important role in the dynamic regulation of UHRF1. When our manuscript was in preparation, Gelato *et al.* reported that binding of PI5P to the Spacer opens the closed conformation of UHRF1 and increases H3K9me3-binding affinity of the TTD^31^. The result suggests that PI5P may facilitate the conformational change of UHRF1 induced by hm-DNA when UHRF1 is recruited to chromatin. In addition, mass-spectrometry analyses have identified several phosphorylation sites (S639, S651, S661) within the Spacer, suggesting that post-translational modification may add another layer of regulation of UHRF1 (refs 32, 37, 39, 40).

It has been well characterized that the SRA of UHRF1 preferentially recognizes hm-DNA through a base-flipping mechanism^18^,^26^,^27^. Our study demonstrates that the Spacer markedly enhances the hm-DNA-binding affinity of the SRA and the deletion of the Spacer impairs heterochromatin localization of UHRF1, indicating that the Spacer is essential for recognition of hm-DNA in the context of full-length UHRF1. Interestingly, variant in methylation 1 (VIM1, a UHRF1 homologue in *Arabidopsis*) contains an equivalent spacer region, which was shown to be required for hm-DNA recognition by its SRA domain^9^,^41^, suggesting a conserved regulatory mechanism in SRA domain-containing proteins. Intriguingly, UHRF2 (the only mammalian homologue of UHRF1) and UHRF1 show very high sequence similarities for all the domains but very low similarity for the Spacer (Supplementary Fig. 7c). Thus, although UHRF2 exhibits the histone- and hm-DNA-binding activities, the difference in the Spacer region may contribute to the functional differences between UHRF1 and UHRF2. This is also consistent with previous finding that UHRF2 is unable to replace UHRF1 to maintain the DNA methylation^14^,^42^,^43^.

One of the key questions in the field of DNA methylation is why UHRF1 contains modules recognizing two repressive epigenetic marks: H3K9me3 (by TTD–PHD) and hm-DNA (by the SRA). Previous studies show that chromatin localization of UHRF1 is dependent on hm-DNA^10^, whereas other studies indicate that histone H3K9me3 recognition and hm-DNA association are both required for UHRF1-mediated maintenance DNA methylation^23^,^34^. However, little is known about the crosstalk between these two epigenetic marks within UHRF1. In this study, we provide an explanation. As shown in the proposed model, recognition of H3K9me3 by full-length UHRF1 is blocked to avoid its miss-localization to unmethylated genomic region, in which chromatin contains H3K9me3 (*K*_D_=4.61 μM) or H3K9me0 (*K*_D_=25.99 μM). We have shown that full-length UHRF1 and SRA–Spacer strongly bind to hm-DNA (0.35 and 0.49 μM, respectively) and the Spacer plays an important role in PCH localization (Fig. 5d). Therefore, genomic localization of UHRF1 is primarily determined by its recognition of hm-DNA, which allows UHRF1 to adopt an open form and promotes its histone tail recognition for proper genomic localization and function. As a result, when SRA–Spacer dissociates from hm-DNA and binds to DNMT1 with a currently unknown mechanism, UHRF1 may keep the complex associated with chromatin through the interaction between TTD–PHD and H3K9me3 (or PHD-H3), and make it possible for DNMT1 to target proper DNA substrate for methylation. This explanation agrees nicely with previous observations and clarifies the importance of coordinate recognition of H3K9me3 and hm-DNA by UHRF1 for maintenance DNA methylation.

UHRF1 is essential for maintenance DNA methylation through recruiting DNMT1 to DNA replication forks during S phase^9^,^10^,^34^,^35^. This function is probably induced by a direct interaction between the SRA and RFTS^DNMT1^ (refs 28, 29, 30) or interaction between DNMT1 and ubiquitylation of histione tail^35^,^44^. Recent study indicates that histone tail association of UHRF1 (by the PHD domain) is required for histone H3 ubiquitylation, which is dependent on ubiquitin ligase activity of the RING domain of UHRF1 (ref. 44). DNMT1 binds to ubiquitylated histone H3 and ubiquitylation is required for maintenance of DNA methylation *in vivo*. In this study, we found that both TTD and PHD are regulated by hm-DNA to recognize histone tail. Thus, the closed form UHRF1 may prevent miss localization of URHF1, whereas only the UHRF1 in open conformation (induced by hm-DNA) could properly binds to histone tail for ubiquitylation and subsequent DNA methylation.

Moreover, structural analyses of DNMT1–DNA^45^,^46^ and SRA–DNA^18^,^26^,^27^ complexes also indicate that it is impossible for DNMT1 to methylate the hm-DNA that UHRF1 binds to because of steric hindrance. In our *in vitro* assays, we could detect interaction between SRA–Spacer and RFTS^DNMT1^, but not the interaction between full-length UHRF1 and RFTS^DNMT1^ (Supplementary Fig. 8a,b and Fig. 5e). The results suggest that UHRF1 adopts multiple conformations. Binding of UHRF1 to hm-DNA may serve as a switch for its recruitment of DNMT1. The S phase-dependent interaction between UHRF1 and DNMT1 (refs 9, 10, 43) suggest that DNMT1 may also undergo conformation changes so that RFTS^DNMT1^ binds to UHRF1 and the catalytic domain of DNMT1 binds to hm-DNA for reaction.

## Methods

### Protein expression and purification

The ubiquitin-like domain (residues 1–133), TTD (residues 134–285), PHD (residues 307–366), TTD–PHD (residues 134–366), SRA (residues 414–617), SRA–Spacer (residues 414–674), Spacer (residues 587–674), RING (residues 675–793), UHRF1 (residues 1–793) and other mutants or truncations of human UHRF1 were sub-cloned in a pGEX-6p-1 derivative vector. The truncated Spacer (residues 627–674) used for NMR analyses was inserted into modified pRSF-Duet-1 vector. All the proteins were expressed in *E. coli* strain BL21 (DE3) and purified as described previously^19^,^20^. In brief, the transformants were grown at 37 °C in 2X YT medium and induced by adding isopropyl-β-D-thiogalactopyranoside (IPTG) to 0.1 mM when the OD_600_ reached 0.6 and further incubated at 15 °C overnight. The cells were harvested and disrupted. After centrifugation, the supernatant of GST-tagged proteins was purified by GST affinity column (GE Healthcare) and the His-tagged truncated Spacer (residues 627–674) was purified by Nickel Nitrilotriacetic Acid affinity chromatography (GE Healthcare). The GST-tagged proteins used for GST pull-down experiment were eluted directly. The fusion proteins were digested with PreScission protease and further purified by ion exchange and gel filtration chromatography. The proteins were concentrated to 5–20 mg ml^−1^ for the following biochemical and structural analyses.

To purify ^15^N- and ^13^C-labelled proteins, the transformants were grown in M9 medium containing ^15^N-labelled NH_4_Cl (1 g l^−1^) and ^13^C-labelled glucose (2 g l^−1^). The isotope-labelled TTD and truncated Spacer were purified as described above.

### Pull-down experiments

For GST pull-down assays, 15 μg GST-tagged proteins were incubated with 40 μg recombinant proteins in 500 μl pull-down buffer (20 mM HEPES-NaOH, pH 8.0, 100 mM NaCl, 5% glycerol and 0.1% Triton X-100) for 1 h at 4 °C. Glutathione resins (GE Healthcare) were washed six times with pull-down buffer then mixed with the proteins for 1 h at 4 °C. After washed three times with pull-down buffer, the bound proteins were analysed by SDS–PAGE Coomassie blue staining. For competitive pull-down experiments: purified proteins were pre-incubated with hemi-methylated-DNA (12 bp, upper strand: 5′-GGGCCXGCAGGG-3′, X=5-methyldeoxycytosine) at the indicated molar ratios for 10 min at 4 °C. For salt concentration-dependent pull-down experiments, the pull-down buffer contains 50, 100 or 150 mM NaCl, respectively.

For histone peptide or hm-DNA pull-down, 1 μg biotinylated histone H3 peptide (residues 1–21) or hm-DNA (12-bp, upper strand 5′-GAGGCXGCCTGC-3′ and lower strand 5′-biotin-GCAGGCGGCCTC-3′, X=5-methyldeoxycytosine) were incubated with 20 μg wild type or mutants of UHRF1 proteins in 500 μl pull-down buffer (20 mM HEPES-NaOH, pH 8.0, 100 mM NaCl, 5% glycerol and 0.1% Triton X-100) for 1 h at 4 °C. The proteins were pre-incubated with hm-DNA (12- bp, upper strand: 5′-GGGCCXGCAGGG-3′, X=5-methyldeoxycytosine) or indicated H3 peptide, or binding buffer as a control, at 1:2 molar ratios for 10 min at 4 °C. Then, 20 μl streptavidin beads were washed six times with pull-down buffer and incubated with the mixture for 1 h at 4 °C. The bound proteins were analysed as described above. The results are summarized in Supplementary Fig. 10.

### ITC measurements

The binding affinity of protein/protein, protein/peptide or protein/DNA was measured by adding 0.05 mM protein in cell and titrated with 0.5 mM protein, peptide or hm-DNA (12 bp, upper strand: 5′-GGGCCXGCAGGG-3′, X=5-methyldeoxycytosine) in the syringe using iTC200 microcalorimeter (GE Healthcare) at 18 °C. For competition ITC experiments: the indicated proteins were pre-incubated with competitive peptide, protein or hm-DNA (at 1:2 molar ratio if not specified) for 10 min followed by ITC measurements as described above. Proteins, DNA and peptides were prepared within ITC buffer containing 10 mM HEPES, pH 8.0, 100 mM NaCl. The data were fitted by software Origin 7.0. All ITC results were summarized in Supplementary Table 2 and raw data were shown in Supplementary Fig. 9.

### NMR titration assay

To determine the mole ratio of TTD versus Spacer peptide (residues 627–674) in the complex for NMR studies, NMR stepwise titration assay was performed at 20 °C in a PBS buffer supplemented with 0.01% NaN_3_, pH 7.4 and 10% D_2_O. The Spacer peptide was added into ^15^N-labelled TTD solution with an increasing molar ratio of TTD/Spacer as follows: 1:0.0, 1:0.2, 1:0.4, 1:0.6, 1:0.8, 1:1.2 and 1:1.5. The ^1^H–^15^N heteronuclear single-quantum correlation (HSQC) spectra of the TTD were collected after each addition.

### NMR spectroscopy and analysis

Two NMR samples were prepared in a mole ratio of 1:1.2 (TTD/Spacer). One is 0.7 mM uniformly ^13^C/^15^N-labelled TTD in complex with unlabelled Spacer peptide in NMR buffer (PBS buffer, 0.01% NaN_3_, pH 7.4 and 10% D_2_O). The other is 0.5 mM ^15^N-labelled Spacer peptide mixed with unlabelled TTD protein. All NMR experiments were performed at 20 °C on a Varian Unity Inova 600 NMR spectrometer equipped with a triple resonances cryoprobe and pulsed field gradients. The standard suite of experiments for assigning the ^1^H, ^13^C and ^15^N backbone, determining the side-chain chemical shifts of the TTD in complex with the Spacer peptide and collecting the Nuclear Overhauser effect (NOE)-based distance restraints were measured^47^, including two-dimensional (2D) ^13^C-edited HSQC and ^15^N-edited HSQC; three-dimensional (3D) HNCA, HNCO, HN(CO)CA, HNCACB, CBCA(CO)NH, ^15^N-resolved HSQC-total correlation spectroscopy (TOCSY) and ^13^C-resolved HSQC-TOCSY in both aliphatic and aromatic regions; ^15^N-resolved HSQC-NOESY; ^13^C-resolved HSQC-NOESY for both aliphatic and aromatic resonances and 2D hbcbcgcdceheA and hbcbcgcdhdA spectra for the correlation of C*β* and H*δ* or H*ɛ* in the aromatic ring that is used for aromatic proton assignment^48^. The NMR signals of bound TTD were assigned according to the previously report^21^. The proton NMR signals of the bound Spacer peptide were assigned by analysing the 2D ^13^C-filtered, ^15^N-filtered and J-resolved NOE spectroscopy (NOESY) and TOCSY spectra recorded for the ^13^C- and ^15^N-labelled protein with the unlabelled Spacer peptide and the 2D ^1^H–^1^H COSY, NOESY and TOCSY spectra recorded for the unlabelled free Spacer peptide, and ^15^N-edited HSQC, 3D ^15^N-resolved HSQC-TOCSY for the ^15^N-labelled Spacer in complex with the TTD protein in the NMR buffer described above, respectively. The intermolecular NOEs between the labelled protein and the unlabelled Spacer peptide were obtained by analysing the 3D ^13^C-F1-edited and ^13^C/^15^N-F3-filtered NOESY spectra. The spectra were processed with the NMRPipe programme^49^ and analysed using Sparky 3 (http://www.cgl.ucsf.edu/home/sparky/).

### Determining the NMR structure

The calculations were performed using a standard simulated annealing protocol implemented in the XPLOR-2.29 programme (NIH version)^50^. The inter-proton distance restraints derived from the NOE intensities were grouped into three distance ranges, namely 1.8–2.9, 1.8–3.5and 1.8–6.0 Å, which corresponds to strong, medium and weak NOEs, respectively. The dihedral angles phi and psi were derived from the backbone chemical shifts (HN, HA, CO and CA) using the programme TALOS^51^. The hydrogen-bond constraints were generated based on the observed NOE pattern between anti-β-sheets in the TTD domain, confirmed by H-D exchange experiments, and used in structural calculation. A total of ten iterations were performed (50 structures in the initial eight iterations). In total, 100 structures were computed during the last two iterations, and the 20 conformers with the lowest energy were used to represent the 3D structures. The conformers of these bundles (TTD in complex with the Spacer peptide) do not violate the following constraints: NOE >0.3 Å and dihedral angle >3^o^. The entire structure statistics were evaluated with PROCHECK^52^ and PROCHECK-NMR^53^ and are summarized in Supplementary Table 3. All of the structure figures were generated using the PyMOL^54^ and MOLMOL programmes^55^.

### Electrophoretic mobility-shift assay

A 6-carboxy-fluorescein (FAM)-labelled primer, 5′-CCATGCGCTGAC-3′, was annealed to a primer 5′-GTCAGXGCATGG-3′ (X=5-methyldeoxycytosine). The hemi-methylated double-strand DNA was used in both electrophoretic mobility-shift assay and FP assays. 50 nM FAM-hm-DNA (1 pmole per lane) was pre-incubated with indicated amount of proteins in reaction buffer (20 mM HEPES, pH 7.5, 100 mM NaCl, 8% glycerol and 1 mM DTT) for 20 min on ice. The samples were subjected to a 10% polyacrylamide gel electrophoresis and run in 0.5 × Tris-borate-EDTA buffer at 100 V for 1 h at 4 °C. The results were visualized on Tanon-5200 Chemiluminescent Imaging System (Tanon Science & Technology Co., Ltd).

### FP measurements

The 12-bp FAM-labelled hm-DNA (as described above) was incubated with increasing amount of indicated proteins for 20 min at 25 °C in reaction buffer containing 20 mM HEPES, pH 7.5, 175 mM NaCl, 8% glycerol and 1 mM DTT. FP measurements were performed at 25 °C on Synergy 4 Microplate Reader (BioTek). The 16-bp and 20-bp FAM-labelled hm-DNA (lower strand: 5′-GTGTCAGXGCATGGCC-3′ and 5′-CCGTGTCAGXGCATGGCCAT-3′, respectively. X=5-methyldeoxycytosine) were used in the FP experiment to test the effect of DNA length on the protein/DNA interaction. All experiments were performed in triplicate. The curves were fitted by GraphPad Prism 5. For salt concentration-dependent FP experiments, the reaction buffer contains 50 mM or 150 mM NaCl, respectively.

### Crystallization and data collection

Crystals of SRA–Spacer in complex with an 18-bp hm-DNA (upper strand: 5′-CATCGTCCCTGCGGGCCC-3′, lower strand: 5′-GGGCCXGCAGGGACGATG-3′. X=5-methyldeoxycytosine) were grown at 18 °C using the hanging drop vapour diffusion method by mixing an equal volume of protein–DNA complex and crystallization buffer containing 12% PEG 3350, 45 mM citric acid/55 mM BIS-TRIS propane (pH 6.9). Protein and hm-DNA were mixed at the molar ratio of 1:1.5 and incubated for 0.5 h on ice before crystallization. Crystals were flash frozen in a cold nitrogen stream at −173 °C. All data sets were collected on beamline BL17U at the SSRF (Shanghai Synchrotron Radiation Facility, China). The data were processed using the programme HKL2000 (ref. 56).

### Structure determination

The structure of SRA–Spacer–hm-DNA complex was determined by molecular replacement using structure of the SRA (PDB:3BI7)^26^ as a searching model. Rotation and translation function searches were performed with the programme PHASER^57^. The model was manually built with COOT^58^. All refinements were performed using the refinement module phenix.refine of PHENIX package^59^. The model quality was checked with the PROCHECK programme^52^ and all structure figures were generated by PyMol^54^.

### Cell culture, transient transfection and images capture

NIH3T3 cells were obtained from the Shanghai Institute of Biochemistry and Cell Biology. Wild type and mutants of UHRF1 were sub-cloned into pEGFP-C1 vector. Transient transfections of NIH3T3 cells were carried out using Lipofectamine 2000 (Invitrogen). The NIH3T3 cells were grown on glass coverslips and harvested in 36 h after transfection. The images were acquired and examined as previously described^34^. Briefly, cells were fixed with 4% paraformaldehyde for 25 min, then washed with PBS three times. Coverslips were mounted with Antifade reagent containing DAPI (Molecular Probes) on slides and examined with a confocal microscopy.

### SAXS measurements

SAXS measurements were performed with Anton Paar SAXSess mc2 instrument with linecolimation and charge-coupled-device detection. The X-ray wavelength was 1.5418 Å (CuKα), the sample to detector was 306.8 mm and the sample slit width was 10 mm. Each sample was prepared in 300 μl solution contained 150 mM NaCl, 10 mM HEPES, pH=8.0, 5 mM DTT and 5% glycerol. For the hm-DNA-bound form, the protein was pre-incubated with hm-DNA (12-bp, upper strand: 5′-GGGCCmCGCAGGG-3′, mC=5-methyldeoxycytosine) at 1:1.2 molar ratio for 10 min on ice. To correct for interparticle interference, the data of protein sample were collected twice and each time for 1 h. The solution containing no protein sample was also tested as background.

The initial data were first processed using SAXSquant and the further analysis with ATSAS software. The SAXS data were only analysed these were collected in the first hour because there was no time effect on the samples. The radius of gyration *R*_g_ was estimated from primus. The distance distribution function *P(r)* was calculated in PCG package. The maximum particle dimension *D*_max_ was estimated from the *P(r)* function as the *r* for which *P(r)*=0.

## Additional information

**Accession codes:** The coordinate and structure factor for the TTD–Spacer complex structure have been deposited in the Protein Data Bank under accession code 5IAY. The chemical shift assignment of TTD–Spacer was deposited with BMRB ADIT-NMR online deposition system under the accession number 30019.

**How to cite this article:** Fang, J. *et al.* Hemi-methylated DNA opens a closed conformation of UHRF1 to facilitate its histone recognition. *Nat. Commun.* 7:11197 doi: 10.1038/ncomms11197 (2016).

## Supplementary Material

Supplementary Information Supplementary Figures 1-10 and Supplementary Table 1-6
