# Supplementary material for: Hemi-methylated DNA opens a closed conformation of UHRF1 to facilitate its histone recognition
Source: Nat Commun. 2016 Apr 5;7:11197. doi: 10.1038/ncomms11197 (PMC4822050; doi:10.1038/ncomms11197)
Supplement: Supplementary Information — Supplementary Figures 1-10 and Supplementary Table 1-6 [file ncomms11197-s1.pdf]

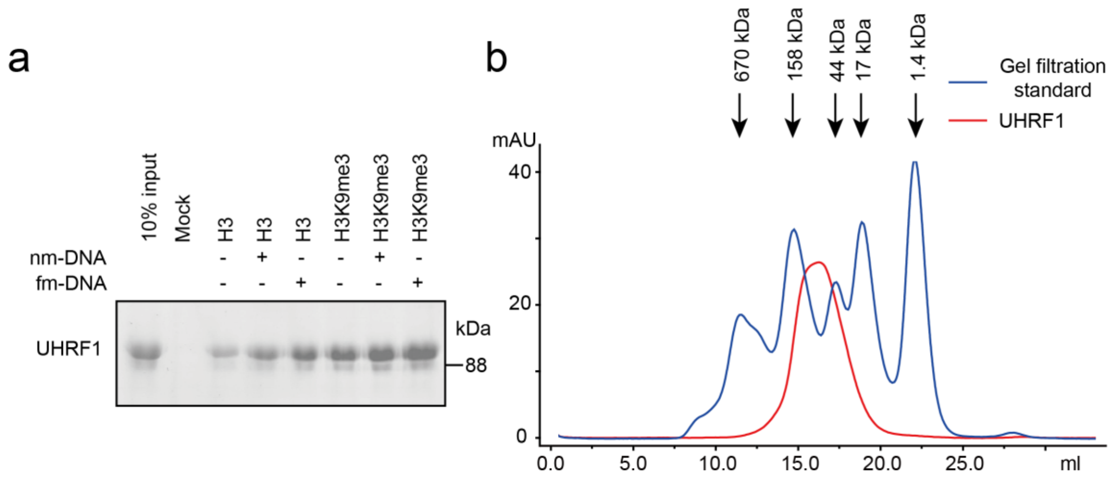

Supplementary Figure 1. The effect of non methylated and fully methylated DNA on the affinity of UHRF1 for histone peptides

(a) Purified full-length UHRF1 was incubated with biotinylated H3 (1-21) or H3K9me3 (1-21) peptides in the presence or absence of nm-DNA (non methylated DNA) or fm-DNA (fully methylated DNA), molar ratio UHRF1: DNA=1:2. The mixture was immobilized onto streptavidin Sepharose beads. The bound proteins were analyzed in SDS-PAGE followed by Coomassie blue staining.

(b) UHRF1 is a monomer in solution. Gel-filtration profile of full-length UHRF1 (MW: 90 kDa) suggests a monomeric form in solution according to the peak position of a standard sample (Superdex 200, 10/300 GL, GE healthcare).

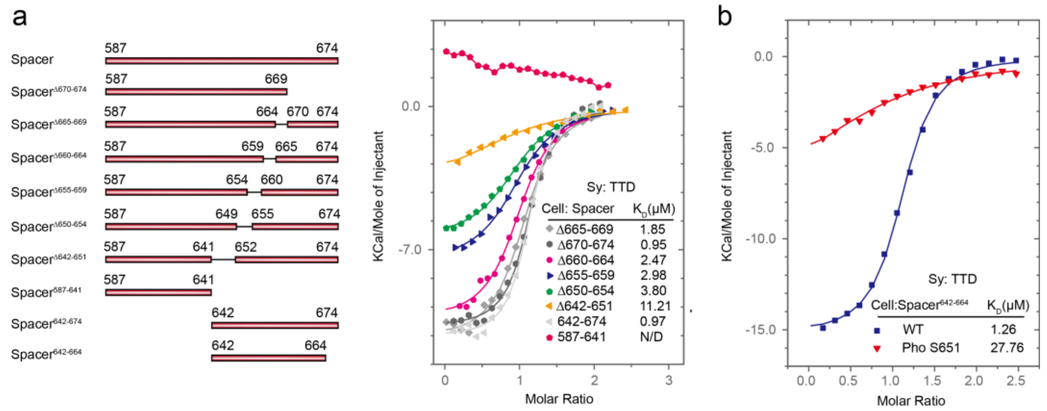

Supplementary Figure 2. The intramolecular interaction between the TTD and the Spacer of UHRF1

(a) Schematic representation of deletions of the Spacer used for ITC measurements (left) and superimposed ITC enthalpy plots for the interaction between the TTD and various Spacer deletions (right).

(b) Superimposed ITC enthalpy plots for the the interaction between the TTD and wild-type or phosphorylated peptide at S651 corresponding to residues 642-664 within the Spacer.

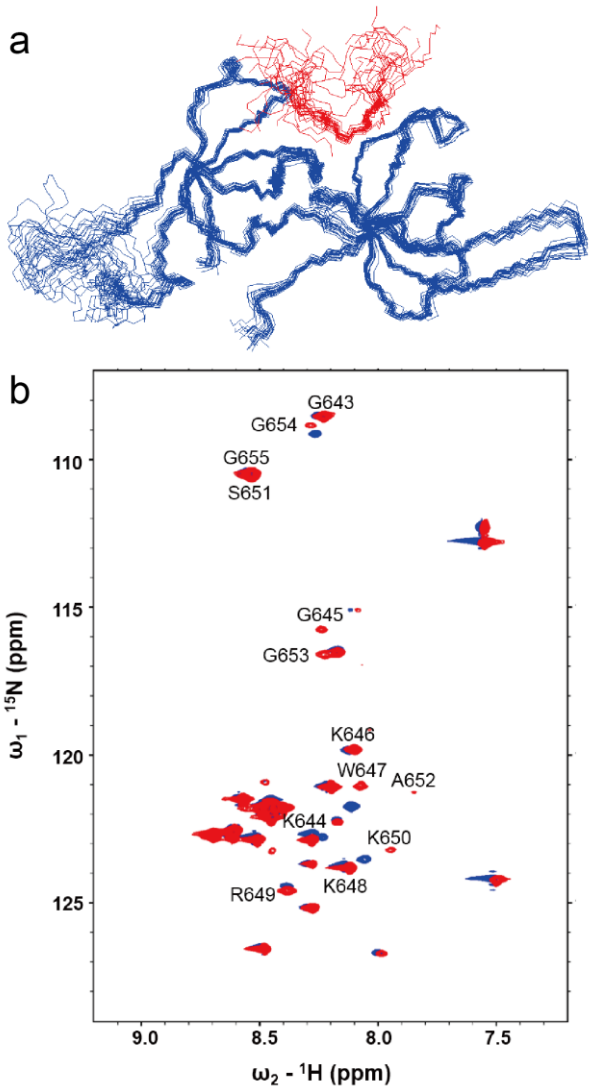

Supplementary Figure 3. The NMR structure of the TTD in complex with the Spacer

(a) Backbone atoms (N, C_α, and C’) of the 20 superposed NMR solution structures of the TTD (blue) in complex with the Spacer peptide (red). The structures were overlaid over the residues 134-285 of the TTD.

(b) The overlay of the ^{1}H–^{15}N HSQC spectra acquired on the Spacer in free state (blue) and in complex with the TTD (red). The ^{1}H–^{15}N cross peak assignments for residues 643-655 are indicated in a one-letter amino acid code.

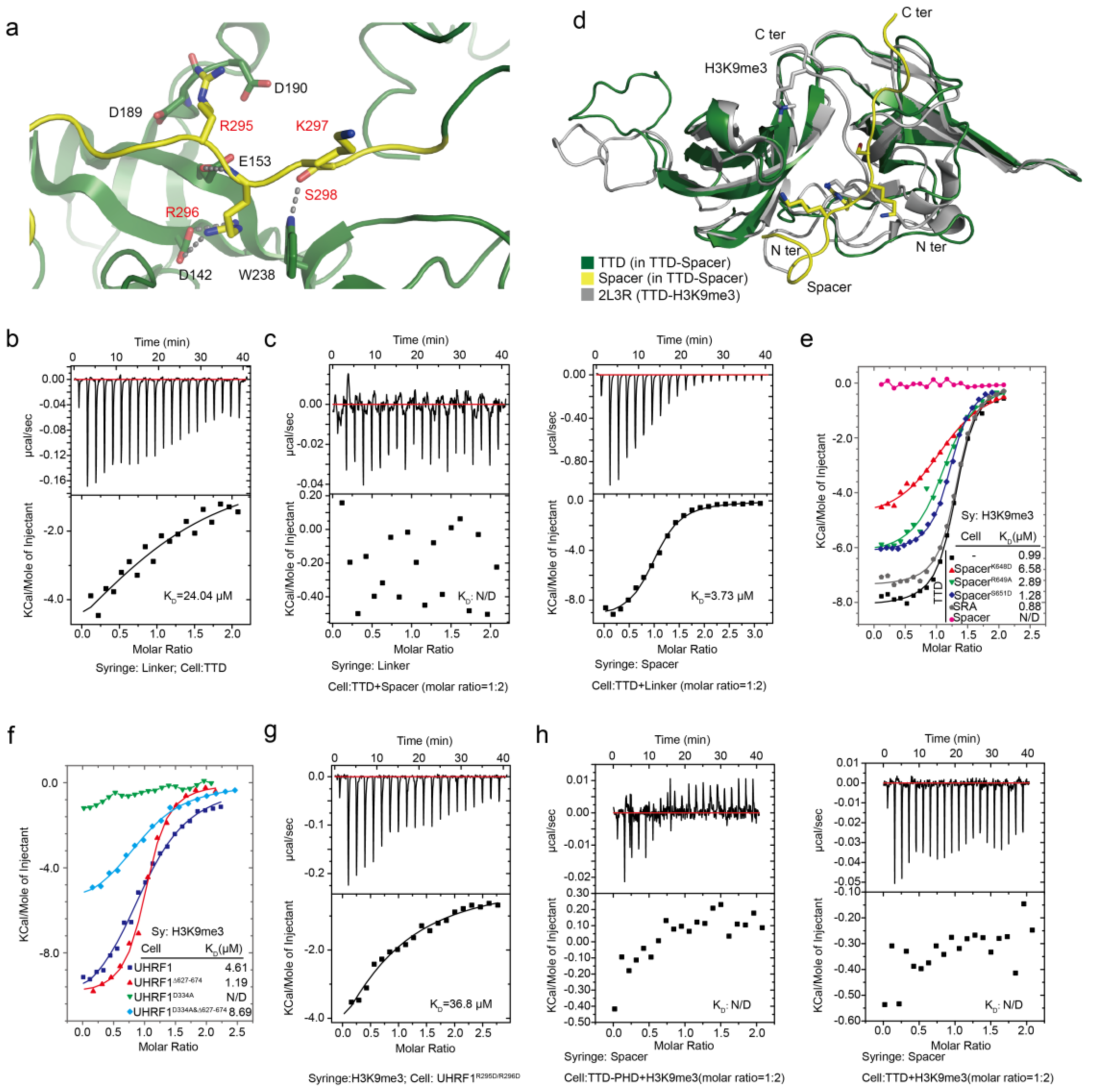

Supplementary Figure 4. The Spacer blocks recognition of H3K9me3 by the TTD through competing with the Linker

(a) Close-up view of the TTD-Linker interaction in TTD-PHD-H3K9me3 complex structure (4GY5.PDB). The TTD and the Linker are colored in green and yellow, respectively. Critical residues for the interaction are shown in stick representation. Hydrogen bonds are indicated as dashed lines.

(b) The ITC titration for the interaction between the Linker and the TTD.

(c) Competition for the TTD binding between the Linker and the Spacer. The ITC titration for TTD-Linker interaction in the presence of the Spacer peptide (left), and

that for TTD-Spacer interaction in the presence of the Linker peptide (right). N/D, no detectable.

(d) Superimposition of the TTD-Spacer and TTD-H3K9me3 (PDB: 2L3R) structures shown in ribbon representations. The TTD-Spacer complex is colored as in Fig. 3a and TTD-H3K9me3 complex is colored in grey. Critical residues for the interaction are shown in stick representation.

(e) Superimposed ITC enthalpy plots for the interaction between H3K9me3 and the TTD in the absence or presence of various Spacer proteins (molar ratio TTD: Spacer=1:2). The TTD and the Spacer are both in the cell for the titration. The SRA serves as a negative control.

(f) Superimposed ITC enthalpy plots for interaction between H3K9me3 and wild-type or mutants of UHRF1. N/D, no detectable.

(g) The ITC titration for the interaction between H3K9me3 and the R295D/R296D mutant of UHRF1.

(h) The ITC measurements for the interaction between the Spacer and TTD-PHD or TTD in the presence of H3K9me3 (molar ratio of TTD-PHD or TTD: H3K9me3=1:2). No detectable interaction was observed in the two titrations.

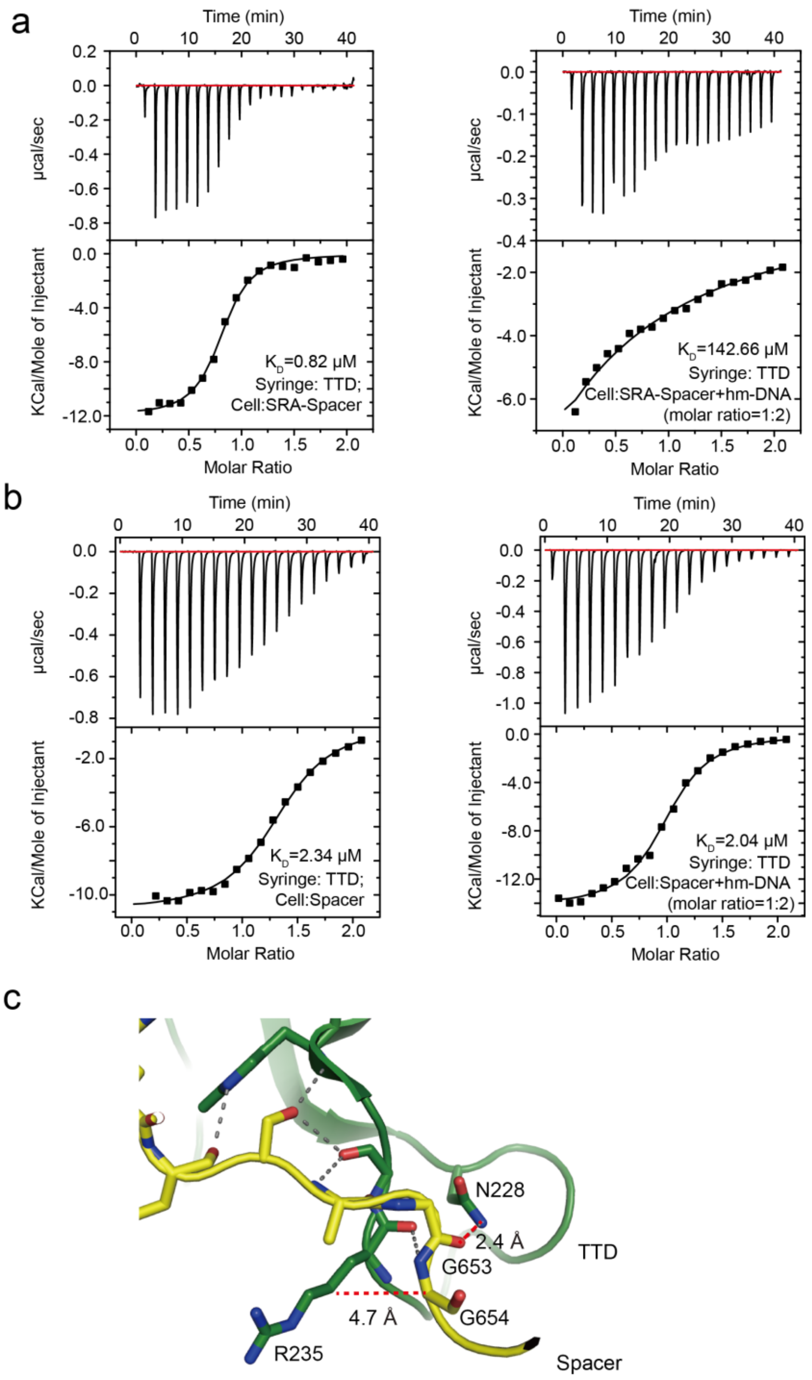

Supplementary Figure 5. Hm-DNA replaces the Spacer from the TTD in a SRA dependent manner.

(a) The ITC titrations for the interaction between the TTD and SRA-Spacer in the absence (left) or presence (right) of hm-DNA (molar ratio SRA-Spacer: hm-DNA = 1:2). The estimated binding affinities (K_D) are indicated.

(b) The ITC titrations for the interaction between the TTD and the Spacer in the absence (left) or presence (right) of hm-DNA (molar ratio Spacer: hm-DNA = 1:2). The estimated binding affinities (K_D) are indicated.

(c) Close-up view of the TTD-Spacer interaction for design of Cysteine mutations of UHRF1 (Fig. 4d). The TTD and the Spacer are colored in green and yellow, respectively. Critical residues for the interaction are shown in stick representation. Hydrogen bonds are indicated as grey dashed lines. The distances (red dashed lines) between selected residues (N228 to G653, R235 to G654 were mutated to Cysteine for the pull-down assay) are short enough for disulfide bond formation. The selected residues are not involved in H3K9me3 recognition (refer to PDB: 4GY5). Note that no other Cysteine residue is located close to this region, which excludes the possible disulfide bond formation with other residues.

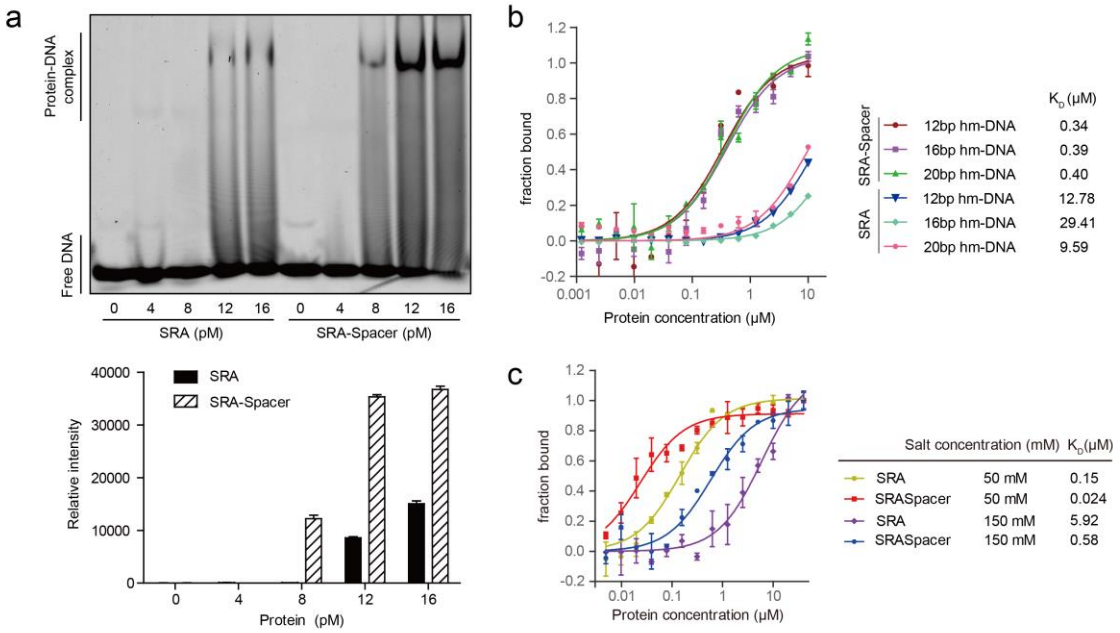

Supplementary Figure 6. The Spacer enhances the DNA-binding activity of the SRA

(a) Electrophoretic Mobility-Shift Assay (EMSA) for the interaction between hm-DNA and the SRA or SRA-Spacer (top). A 12-bp FAM-hm-DNA was incubated with increasing amount of SRA or SRA-Spacer proteins. Protein concentrations are indicated. The results were quantified by band densitometry (bottom). Error bars, s.d. for triplicate experiments.

(b) Superimposed fluorescence polarization (FP) for hm-DNA-binding affinities of SRA or SRA-Spacer. Various lengths of hm-DNA were used.

(c) Superimposed fluorescence polarization (FP) for hm-DNA-binding affinities of SRA or SRA-Spacer in a salt concentration-dependent manner. The concentrations of NaCl are indicated.

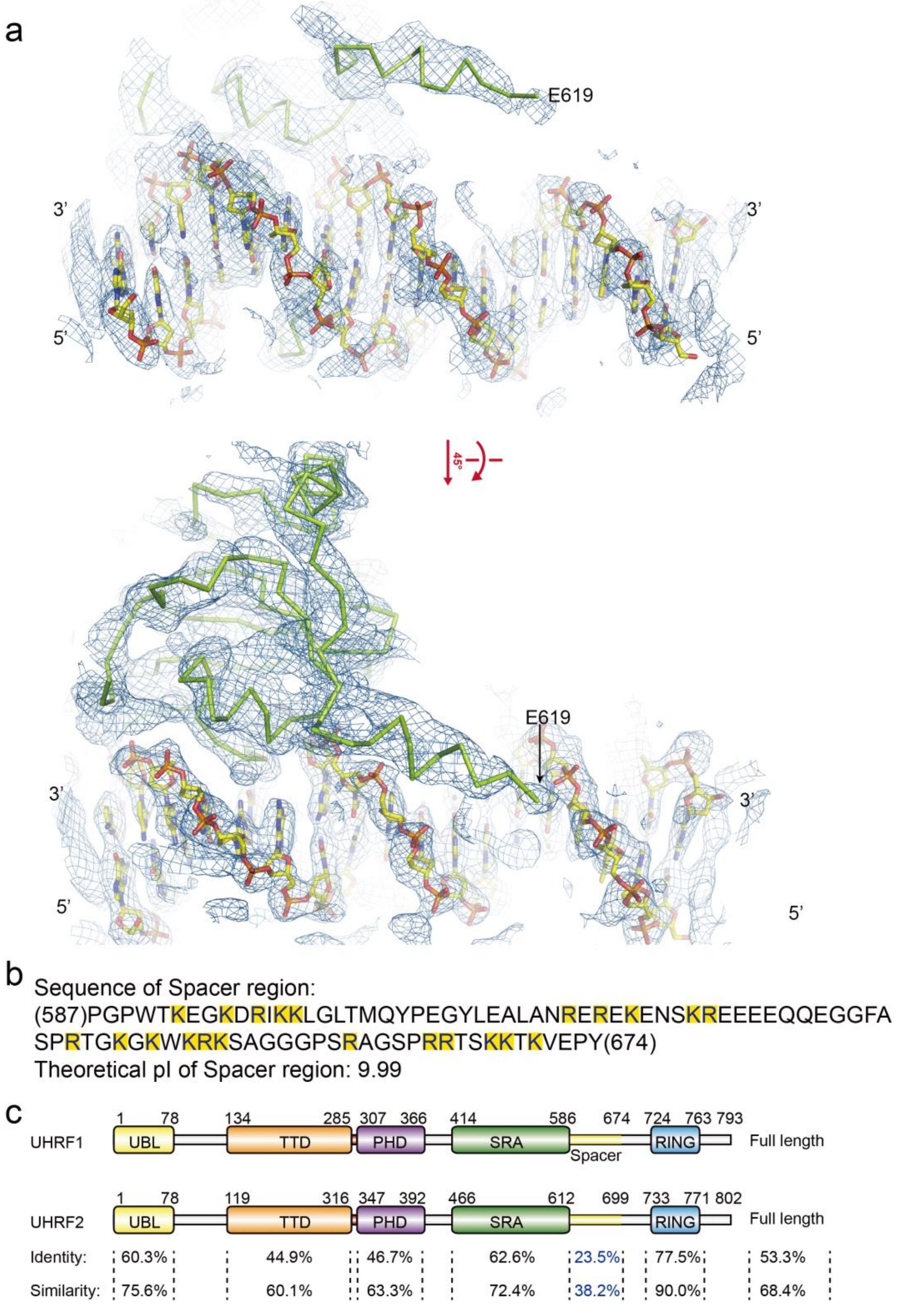

Supplementary Figure 7. Crystal structure of SRA-Spacer bound to hm-DNA

(a) $2F_{observed}$-$F_{calculated}$ map for SRA-Spacer-hm-DNA structure. The maps were calculated at 3.15 Å and contoured at 1.0 σ. Only main-chains are shown for

simplicity. Note that residues 617-674 were not built because of lacking the electron density.

(b) Primary sequence of the Spacer with the basic residues highlighted with yellow background.

(c) Sequence identities and similarities between domains or regions of human UHRF1 and UHRF2. Note that the Spacer is less conserved compared to other domains.

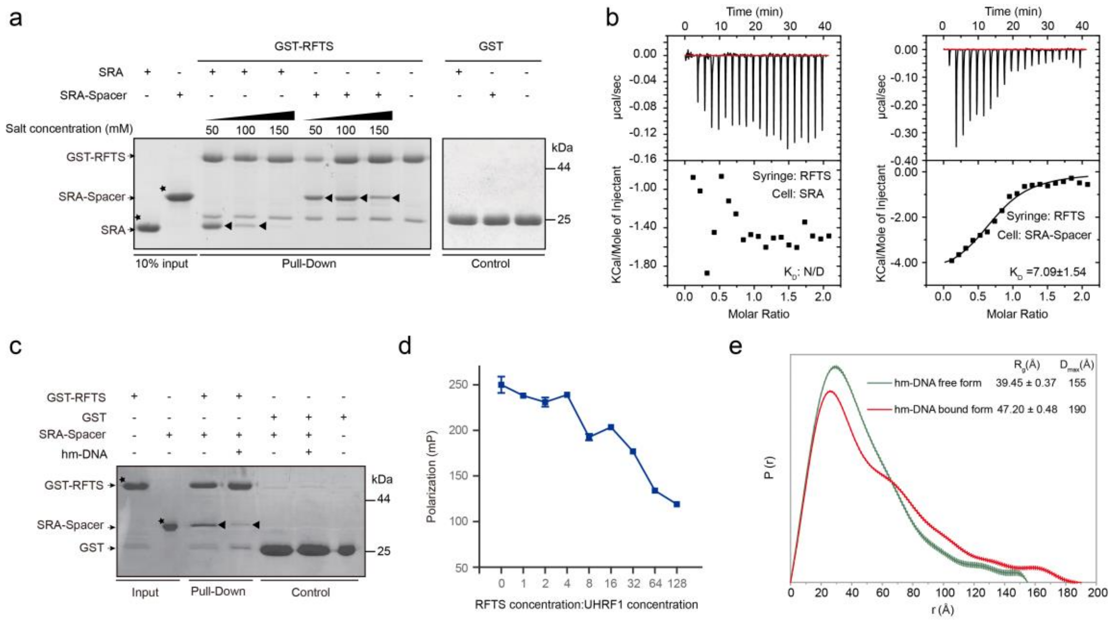

Supplementary Figure 8. The Spacer facilitates the interaction between UHRF1 and DNMT1

(a) Interactions between $RFTS^{DNMT1}$ and the SRA or SRA-Spacer in a salt concentration-dependent manner. GST pull-down experiments were performed as described in Fig. 2a. The concentrations of NaCl are indicated.

(b) ITC titrations for the interaction between $RFTS^{DNMT1}$ and the SRA or SRA-Spacer.

(c) The GST pull-down assay for the interaction between GST-tagged $RFTS^{DNMT1}$ and SRA-Spacer in the presence or absence of hm-DNA (molar ratio SRA-Spacer: hm-DNA = 1:2). The experiments were performed as described in Fig. 2a.

(d) Fluorescence polarization measurement for UHRF1-hm-DNA complex formation in the presence of $RFTS^{DNMT1}$. A mixture of UHRF1 (100 pM) and a 12-bp FAM-hm-DNA (0.5 pM) was pre-incubated for 30 min. Increasing amount of $RFTS^{DNMT1}$ was added to the mixture before the measurements. The levels of

UHRF1-hm-DNA complex formation are represented by fluorescence polarization response (in milli-polarization units, mP).

(e). SAXS analysis is shown for the hm DNA free form (green) or bound form (red) of TTD-PHD-SRA-Spacer.

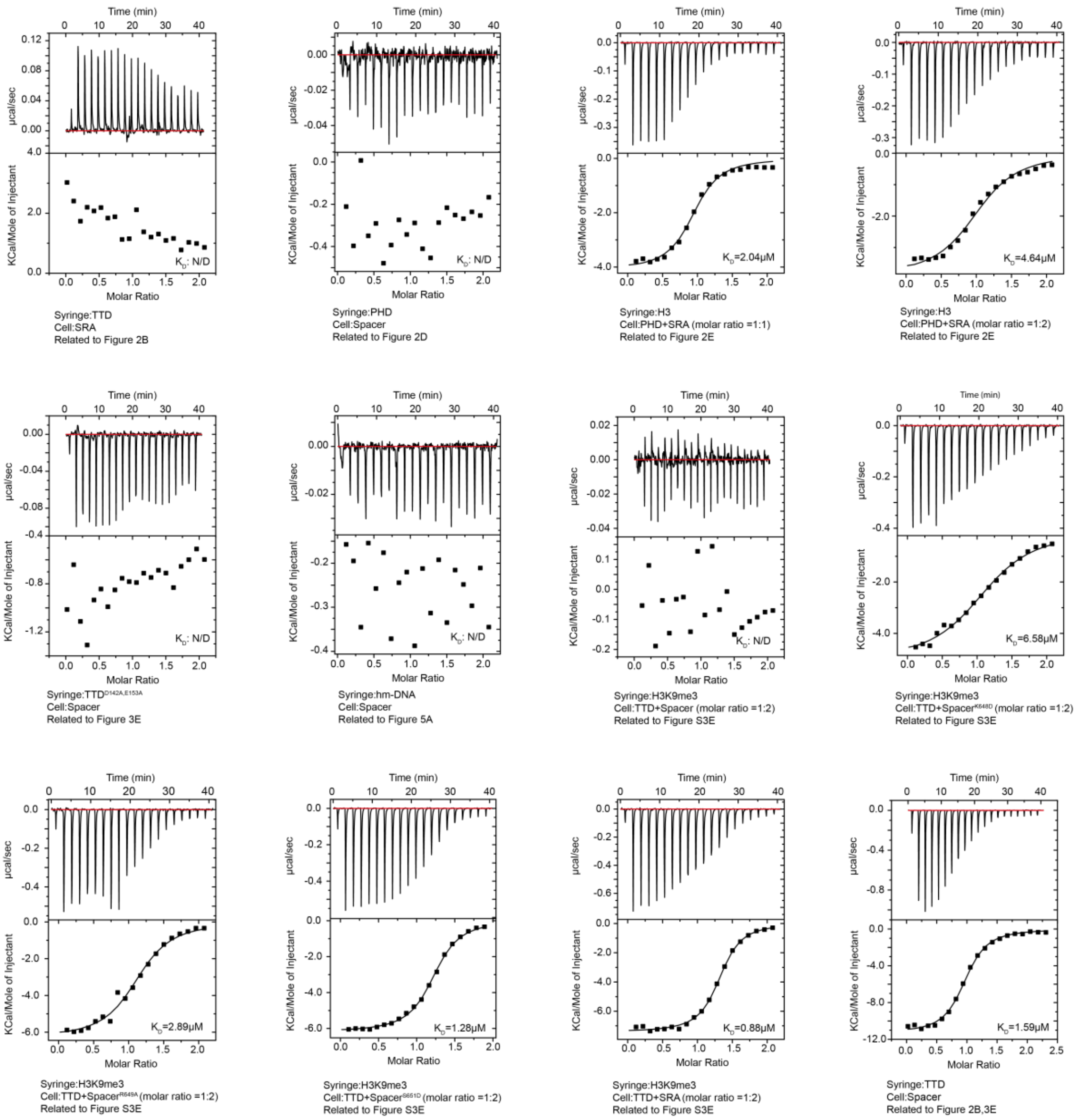

Supplementary Figure 9. A collection of raw data for ITC titrations performed in this work.

All the parameters are summarized in Supplementary Table 2.

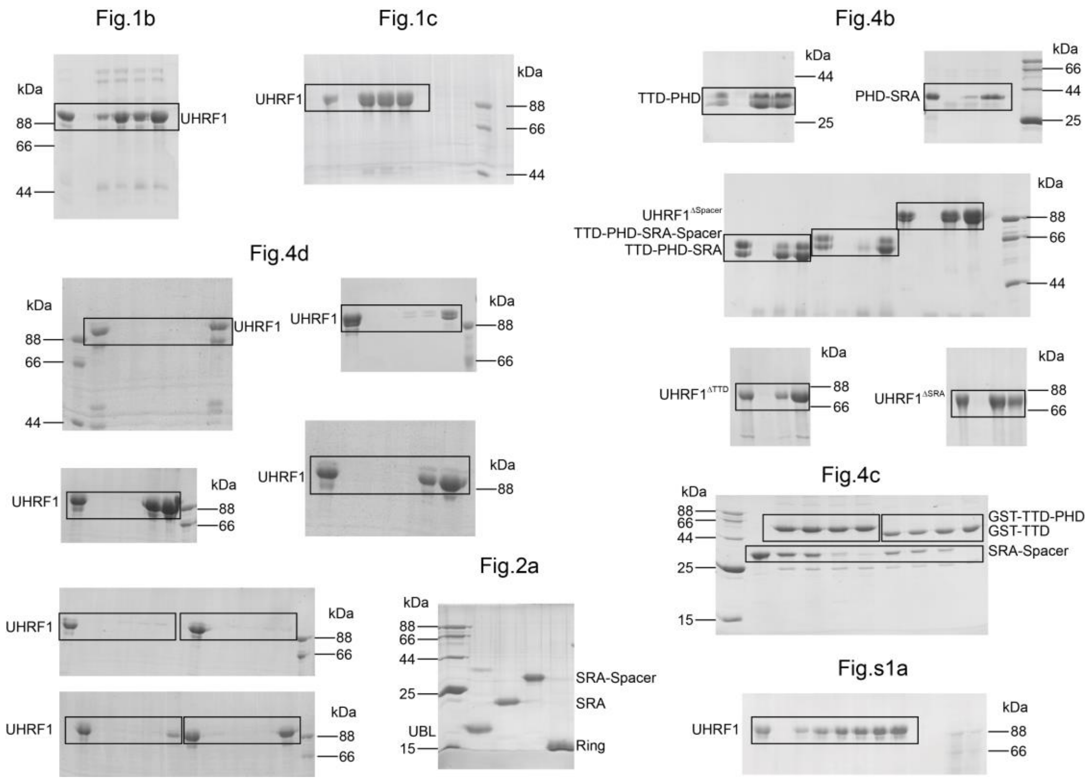

Supplementary Figure 10. Full uncropped figures of pull-down experiments.

Supplementary Table 1. Histone peptides used in this work

Peptide	Boundary	Sequence (amino acid)
H3K9me0	1-17	ARTKQTARKSTGGKAPR
H3K9me3	1-17	ARTKQTARK(me3)STGGKAPR
Biotin- H3K9me0	1-21	Biotin-ARTKQTARKSTGGKAPRKQLA
Biotin- H3K9me3	1-21	Biotin-ARTKQTARK(me3)STGGKAPRKQLA

Supplementary Table 2. Summary of Isothermal Titration Calorimetry (ITC)

		**K_D (μM)**	**ΔH (cal/mol)**	**ΔS (cal/mol/deg)**	**N**
**H3K9me3**					
UHRF1		4.61±0.47	-4.646E4±1233	-57.3	1.05±0.0196
TTD	-	0.99±0.09	-8136±71.52	-0.455	1.28±0.00794
	Spacer wt	N/D	N/D	N/D	N/D
	Spacer^{K648D}	6.58±0.74	-5049±118.2	6.41	1.17±0.0184
	Spacer^{R649A}	2.89±0.53	-6300±166.7	3.79	1.13±0.0218
	Spacer^{S651D}	1.28±0.07	-6184±32.11	5.73	1.21±0.00447
	SRA	0.88±0.07	-7415±53.15	2.25	1.29±0.00646
TTD-PHD	-	0.15±0.02	-1.260E4±95.03	-12.0	0.990±0.00526
	Spacer(1:2)	1.63±0.08	-8455±55.98	-2.54	0.993±0.00481
UHRF1^{D334A}		N/D	N/D	N/D	N/D
UHRF1$^{\Delta 627\text{-}674}$		1.19±0.17	-1.004E4±185.3	-7.32	0.988±0.0133
UHRF1$^{D334A\&\Delta 627\text{-}674}$		8.69±0.69	-6173±204	1.96	0.979±0.02
**H3**					
PHD		0.77±0.08	-6377±62.92	6.08	0.935±0.00653
UHRF1		25.99±7.01	-7147±738	-3.41	1.18±0.063
PHD+SRA(1:1)		2.04±0.31	-4093±88.16	12.0	0.938±0.0149
PHD+SRA(1:2)		4.64±0.73	-3890±116.8	11.1	1.05±0.0224
PHD-SRA		5.79±0.99	-5535±173.7	5.03	1.19±0.0254
**Spacer**					
PHD		N/D	N/D	N/D	N/D
TTD^{D142A,E153A}		N/D	N/D	N/D	N/D
TTD(fg4g)		1.48±0.18	-1.614E4±297.4	-28.7	0.991±0.0134
TTD-PHD		10.68±2.01	-6561±411.6	0.285	0.887±0.0379
TTD-PHD^{RR295,296DD}		2.69±0.38	-1.399E4±290.7	-22.5	0.944±0.0158
TTD+Linker		3.73±0.38	-9581±182.9	-8.08	1±0.016
**PHD**					
SRA		26.71±12.05	-3506±979.2	9.34	0.823±0.16
**TTD**					
Spacer		1.59±0.13	-1.13 E4±116.7	-12.3	0.95±0.00786

Spacer$^{587-641}$	N/D	N/D	N/D	N/D
Spacer $^{\Delta 642-651}$	11.21±2.38	-3833±425.9	9.58	0.891±0.0733
Spacer$^{642-674}$	0.97±0.09	-1.128 E4±111	-11.2	1.09±0.00765
Spacer$^{\Delta 650-654}$	3.80±0.33	-6475±107.1	2.57	0.967±0.0122
Spacer$^{\Delta 655-659}$	2.98±0.30	-7634±156.1	-0.906	0.988±0.0147
Spacer$^{\Delta 660-664}$	2.47±0.21	-1.046 E4±150.4	-10.2	1.01±0.00976
Spacer$^{\Delta 665-669}$	1.85±0.16	-1.139 E4±133.1	-12.9	1.06±0.00978
Spacer$^{\Delta 670-674}$	0.95±0.12	-1.079 E4±139.2	-9.46	1.1±0.0102
Spacer^{R649A}	21.40±1.93	-8277±352.0	-7.04	1.06±0.0256
Spacer^{K648D}	5.94±0.87	-8450±365.9	-5.07	0.659±0.0209
Spacer^{S651D}	8.81±0.60	-1.237 E4±321.1	-19.3	0.998±0.0189
Spacer^{S639D}	1.15±0.13	-1.125E4±153.8	-11.4	1.03±0.0102
Spacer^{S666D}	1.26±0.12	-1.219E4±159.8	-14.9	1.08±0.0104
Spacer$^{642-664}$	1.26±0.10	-1.528E4±166.4	-25.5	1.08±0.0086
Spacer$^{642-664\ PhoS651}$	27.76±4.72	-9098±1371	-10.3	0.871±0.0996
SRA	N/D	N/D	N/D	N/D
SRA-Spacer	0.82±0.12	-1.199E4±224.9	-13.3	0.788±0.0108
SRA-Spacer +hm-DNA	142.66±68.75	-6.18E5±1.69E7	-2.11E3	0.0326±0.885
Linker	24.04±8	-7850±3156	-5.83	1.21±0.2
**hm-DNA**				
SRA	25.12±5.06	-2648±221.3	12.0	1.25±0.0495
SRA-Spacer	1.75±0.30	-4355±90.57	11.4	1.15±0.0175
Spacer	N/D	N/D	N/D	N/D
**RFTS**				
SRA	N/D	N/D	N/D	N/D
SRA-Spacer	7.09±1.54	-4721±317.0	7.43	0.749±0.0361
**Linker**				
TTD+Spacer	N/D	N/D	N/D	N/D

N/D represents not detectable.

Supplementary Table 3. NMR structural statistics for the TTD bound to the Spacer

**Distance and dihedral restraints**	
Total	3128
Intra-residue (i-j=0)	633
Sequentia l(|i-j|=1)	902
Medium range (1<|i-j|<5)	528
Long range (|i-j|>5)	1061
Intermolecular	19
Dihedral restraints	184
Hydorgen bonds	112
**Structural statistics**	
**Rms Deviations versus the mean structure (Å)**	
All backbone atoms	1.42 ± 0.40
All heavy atoms	1.69 ± 0.33
Backbone atoms (secondary structure)	0.76 ± 0.21
Heavy atoms (secondary structure)	1.15 ± 0.14
**R.m.s. deviations from the experimental restraints**	
NOE distances (Å)	0.014 ± 0.00082
Dihedral angles (°)	0.67 ± 0.034
**R.m.s. deviations from idealized geometry**	
Bonds (Å)	$0.0013 \pm 3.73\times10^{-5}$
Angles (°)	0.26±0.0063
Impropers (°)	0.26 ± 0.0068
**Ramachandran analysis**	
Residues in most favored regions	84.8%
Residues in additonally allowed regions	13.1%
Residues in generously allowed regions	2.1%
Residues in disallowed regions	0.0%

Supplementary Table 4. Fluorescence Polarization measurements for the interaction between hm-DNA and UHRF1

	K_D (μM)
**FAM-12bp-HM-DNA**	
SRA-Spacer-642	6.11±1.15
SRA-Spacer-652	1.87±0.33
SRA-Spacer-661	0.38±0.06
SRA-Spacer	0.49±0.08
$UHRF1^{\Delta 627-674}$	4.94±1.09
UHRF1	0.35±0.07
SRA	9.23±3.07
$UHRF1^{\Delta TTD}$	0.49±0.08
$UHRF1^{\Delta SRA}$	N/D

N/D represents not detectable.

Supplementary Table 5. Fluorescence Polarization measurements for hm-DNA-binding affinities for the SRA or SRA-Spacer

	K_D (μM)		
	**FAM-12bp-HM-DNA**	**FAM-16bp-HM-DNA**	**FAM-20bp-HM-DNA**
SRA	12.78±2.07	29.41±9.74	9.59±4.78
SRA-Spacer	0.34±0.09	0.39±0.07	0.40±0.07
	**FAM-12bp-HM-DNA**		
SRA	0.15±0.018 (50 mM NaCl)	5.92±1.32 (150 mM NaCl)	
SRA-Spacer	0.024±0.0037 (50 mM NaCl)	0.58±0.098(150 mM NaCl)	

Supplementary Table 6. Crystallographic data and structure refinement statistics

**Data collection and reduction statistics**	
Protein	SRA bound to 18-bp hm-DNA
Beamline	SSRF BL17U
Wavelength (Å)	0.97927
Resolution (Å)	50-3.15 (3.26- 3.15)
Space group	P43
Unit cell (Å)	85.7, 85.7, 167.7
	90, 90, 90
Completeness(%)[a]	99.72 (99.25)
R_{merge} (%)	0.126 (0.786)
I/σ (I)	8.27 (2.42)
Total reflections	159694
Unique reflections	21182
Redundancy	7.5 (7.4)
**Refinement**	
Resolution range (Å)	50-3.15 (3.26 - 3.15)
R_{work}/R_{free} (%)[b]	22.71/27.26
Number of atoms	4695
Protein	3227
DNA	1468
R.m.s deviations	
Bond lengths (Å)	0.010
Bond angles (º)	1.67
Average B-factor (Å^2)	93.40
Protein	101.10
DNA	76.60

[a] The values for the data in the highest resolution shell are shown in parentheses.

[b] $R_{free} = \sum_{Test}||F_{obs}| - |F_{calc}||/\sum_{Test} |F_{obs}|$, where "Test" is a test set of about 5% of the total reflections randomly chosen and set aside prior to refinement for the complex.
